# Zeolitic imidazolate framework-8: a versatile nanoplatform for tissue regeneration

**DOI:** 10.3389/fbioe.2024.1386534

**Published:** 2024-04-09

**Authors:** Zhixin Li, Yinjin Shao, Youwen Yang, Jun Zan

**Affiliations:** ^1^ Department of Rehabilitation, Ganzhou People’s Hospital, Ganzhou, China; ^2^ Institute of Additive Manufacturing, Jiangxi University of Science and Technology, Nanchang, China

**Keywords:** zeolitic imidazolate framework, tissue regeneration, versatile nanoplatform, biocompatibility, drug delivery

## Abstract

Extensive research on zeolitic imidazolate framework (ZIF-8) and its derivatives has highlighted their unique properties in nanomedicine. ZIF-8 exhibits advantages such as pH-responsive dissolution, easy surface functionalization, and efficient drug loading, making it an ideal nanosystem for intelligent drug delivery and phototherapy. These characteristics have sparked significant interest in its potential applications in tissue regeneration, particularly in bone, skin, and nerve regeneration. This review provides a comprehensive assessment of ZIF-8’s feasibility in tissue engineering, encompassing material synthesis, performance testing, and the development of multifunctional nanosystems. Furthermore, the latest advancements in the field, as well as potential limitations and future prospects, are discussed. Overall, this review emphasizes the latest developments in ZIF-8 in tissue engineering and highlights the potential of its multifunctional nanoplatforms for effective complex tissue repair.

## Graphical Abstract

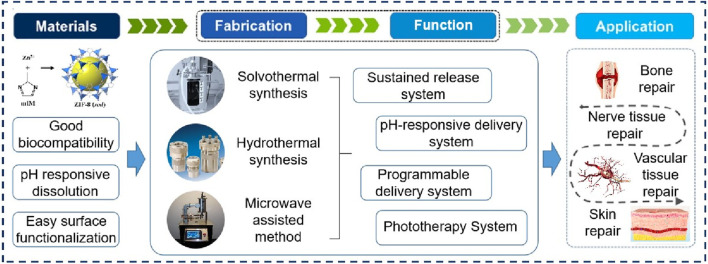

## 1 Introduction

Tissue defects and regeneration have long been a global challenge, prompting continuous exploration and research by humankind (Christman, 2019; Yin et al., 2020; Wei et al., 2021). In recent years, the risks associated with tissue injuries have become increasingly complex and diverse due to factors such as population aging, frequent traffic accidents, and changes in physiological diseases (Huynh et al., 2019; Hoeffel et al., 2021). In China alone, statistics indicate that over 40 million patients suffer from tissue damage, with bone tissue defects, soft tissue injuries, and nerve damage being the primary concerns (Li and Ouyang, 2022). Unfortunately, many patients are unable to receive timely and effective treatments, often resorting to conservative measures or even amputation due to the scarcity of suitable bone biomaterials (Yang H. et al., 2020; Li and Felländer-Tsai, 2021). Therefore, it is both clinically significant and socially beneficial to explore biomaterials with excellent properties to address these challenges.

Over the past three decades, autologous and allogeneic transplantation have been sequentially employed to treat tissue defects (Jiang et al., 2021a; Li et al., 2021). However, the limited availability of autologous donors and the potential for immune rejection with allogeneic transplants have hindered their widespread application (Wang et al., 2019; Schmidt, 2021). Consequently, there is a pressing need for the development of a comprehensive, safe, and effective treatment approach. Artificial tissue transplantation, first proposed in 1994, has garnered significant attention in the field of tissue regeneration (Savolainen et al., 1994; Zuo et al., 2016). These transplants utilize a wide range of matrix materials, including biometals, bioceramics, and biopolymers, allowing for countless combinations and diverse properties (Tiffany et al., 2019; Yao et al., 2022; Feng et al., 2023a). Additionally, these biomaterials exhibit excellent biocompatibility and are not prone to immune rejection (Guo et al., 2019; Wang et al., 2022; Feng et al., 2023a). Notably, considering the diverse locations and causes of tissue damage, these biomaterials must not only possess inherent biocompatibility and mechanical strength but also incorporate special functions such as antibacterial properties (Xu et al., 2020; Zhao et al., 2022), anti-inflammatory effects (Zhu et al., 2021; Wu et al., 2023), and anti-tumor capabilities (Wang et al., 2020; Jiang et al., 2021b). Consequently, the exploration of functionally enhanced artificial tissues has become a prominent research focus in the past decade.

From a material perspective, functional artificial tissue involves the integration of functional nanoparticles into a biomaterial matrix. The key challenge lies in constructing suitable nanoparticles for specific environments (Anju et al., 2020; Wei et al., 2021). Metal-organic frameworks (MOFs) have recently emerged as materials with limitless potential, thanks to their unique structure and diverse functions (Cai et al., 2021; Wu et al., 2021; Xu et al., 2021). Indeed, MOFs have defied numerous expectations in the world of crystalline porous materials. They possess a high surface area (ranging from 1,000 to 9,800 m^2^/g), large porosity (over 55% with pore sizes ranging from 5 to 90 Å), and excellent chemical and thermal stability (up to 350°C–600°C) (Bagi et al., 2021; Guillerm and Eddaoudi, 2021). Moreover, the availability of multiple building units enables the creation of exciting new functions (Bagi et al., 2021; Guillerm and Eddaoudi, 2021). As of 2021, the Cambridge Structural Database contained over 90,000 MOF structures (Moosavi et al., 2020). Leveraging their unique structure and wide variety, MOFs have found applications in tissue engineering, medicine, and bioimaging (Du et al., 2021; Xue et al., 2021; Fardjahromi et al., 2022).

Among the various MOF materials, zeolitic imidazolate framework-8 (ZIF-8) has garnered significant interest in the field of tissue regeneration (Yang M. et al., 2022; Doustdar and Ghorbani, 2022). ZIF-8 is formed by zinc ions (Zn^2+^) and dimethyl imidazole (Nordin et al., 2014; Wu et al., 2022), with Zn^2+^ being one of the most abundant transition metals in biology and the imidazole group being an essential component of amino acids (Roth and Breaker, 1998; Stefanidou et al., 2006). This unique composition gives ZIF-8 good biocompatibility compared to other MOFs, making it well-suited for tissue repair. Additionally, ZIF-8 exhibits stable chemical and thermal properties in aqueous environments (Tian et al., 2014), but can decompose under acidic conditions (Zhu et al., 2020; Zan et al., 2023). This pH-responsive behavior offers opportunities for specific applications in humoral environments, such as pH-controlled delivery and release in inflammatory settings (Feng et al., 2021). Furthermore, the framework structure of ZIF-8 enables the loading of functional nano drugs for targeted treatment or serves as an ideal self-sacrificial template for fabricating hollow nanomaterials (Kaur et al., 2017; Zheng et al., 2017). So far, ZIF-8 and its derivatives have found wide applications in tissue engineering, including drug delivery platforms for precise targeted drug delivery, the construction of phototherapy nanoplatforms, and multifunctional antibacterial platforms.

The exceptional potential and minimal toxicity of ZIF-8 make it a promising candidate for enhancing tissue scaffold performance in repairing and reconstructing injured tissue. This review focuses on recent advancements in tissue regeneration, specifically highlighting the role of ZIF-8 in constructing multifunctional nanoplatforms and its effectiveness in tissue regeneration scaffolds. It provides in-depth research on the synthesis parameters, techniques, and analysis of the physicochemical and biological properties of ZIF-8 derivatives. Notably, this review stands out as there is currently no similar literature emphasizing the field of tissue regeneration. As a result, this work serves as a valuable guide for researchers, offering insights and opinions in this area of study.

## 2 Synthesis and performance of ZIF-8

ZIF-8 is a porous material with a sodalite (SOD) topology, characterized by interconnected six-membered windows (Liang et al., 2018; Gao et al., 2022). It consists of zinc ions coordinated with 2-methyl imidazolate ligands. The biomedical potential of ZIF-8 primarily stems from its high porosity, pH-responsive behavior, drug adsorption capabilities, and surface functionalization possibilities (Bieniek et al., 2021). Continuous innovation in material science has led to the development of green synthesis methods and size optimization techniques for ZIF-8, prompting researchers to explore various preparation approaches. Precise control over ZIF-8 synthesis is crucial for developing more stable, powerful, and structurally complex materials. Additionally, understanding the physicochemical properties, cytotoxicity, and optimal release kinetics of ZIF-8 is essential for its application in tissue engineering. This chapter provides a comprehensive discussion on the synthesis and performance of ZIF-8.

### 2.1 ZIF-8 fabrication technologies

#### 2.1.1 Solvothermal synthesis

Solvothermal synthesis is the most commonly used method for obtaining high yields and purity of ZIF-8 (Cravillon et al., 2012). The solvothermal method was initially reported by Park et al., in 2006, and since then, it has become a prominent approach for developing high-quality ZIF-8 (Park et al., 2006). This method is simple and effective, involving the dispersion of zinc salt and 2-methylimidazole in an organic solvent to form a composite solution, which is then heated at a specific temperature. Under the influence of the solvent and heat, the 2-methylimidazole reacts with zinc ions, forming crystal nuclei of ZIF-8. Over time, these nuclei grow until neutral 2-methylimidazole is observed. Currently, the organic solvent commonly used for the synthesis of ZIF-8 include, but are not limited to, methanol (Malekmohammadi et al., 2019), N,N-dimethylformamide (DMF) (Santoso et al., 2021) and N. N-diethylformamide (DEF) (Reif et al., 2019), etc.

In their study, Ahn et al. utilized DMF as the organic solvent and employed the solvothermal method to prepare rhombic ZIF-8 particles with an average size ranging from 150 to 250 μm (Lee et al., 2015). In a separate study conducted by Wiebcke et al., in 2009, methanol was used as the organic solvent to synthesize nano-sized ZIF-8 particles at room temperature (Cravillon et al., 2009). The results showed that the ZIF-8 particles exhibited a regular particle morphology, a narrow size distribution (with an average particle size of approximately 46 nm), and good thermal stability, up to approximately 200 °C. Edit and colleagues utilized acetic acid as a polar solvent to synthesize uniform ZIF-8 particles at room temperature (Santoso et al., 2021). Compared to the ZIF-8 synthesized using DMF, the particles produced through this method had a more uniform distribution and smaller size (with an average particle size of approximately 65 nm). Furthermore, this method effectively enhanced the mesoporosity of the ZIF-8 particles, resulting in a larger pore diameter (2.76 nm), a higher mesopore volume (0.166 cc/g), and a moderate surface area (500 m^2^/g).

Although solvothermal synthesis is known for producing high-quality ZIF-8, it is worth noting that this approach can be energy-intensive and time-consuming. Additionally, the synthesis process often requires the use of excessive amounts of organic solvents, which can result in significant pollution and potential harm to biosafety (Pan et al., 2022). Many organic solvents are known to be toxic to the human body, which poses a significant challenge for the application of ZIF-8 in tissue engineering, unless careful consideration is given to the removal or avoidance of these solvents. In summary, due to these concerns, solvothermal synthesis may not be suitable for the preparation of ZIF-8 for medical applications.

#### 2.1.2 Hydrothermal method

In addition to solvothermal synthesis, hydrothermal synthesis is another commonly used method to obtain high purity ZIF-8 (Munn et al., 2015; Butova et al., 2017). This aqueous synthesis method effectively addresses the issue of using organic solvents by solely relying on water as the polar solvent, resulting in the formation of well-defined lattice crystals. Furthermore, hydrothermal synthesis offers the advantage of operating at room temperature, eliminating the need for high-temperature conditions and thereby increasing production efficiency (Pan and Lai, 2011; Shi et al., 2011). As a result, hydrothermal synthesis has emerged as an attractive approach for the environmentally friendly and safe production of ZIF-8.

**FIGURE 1:**
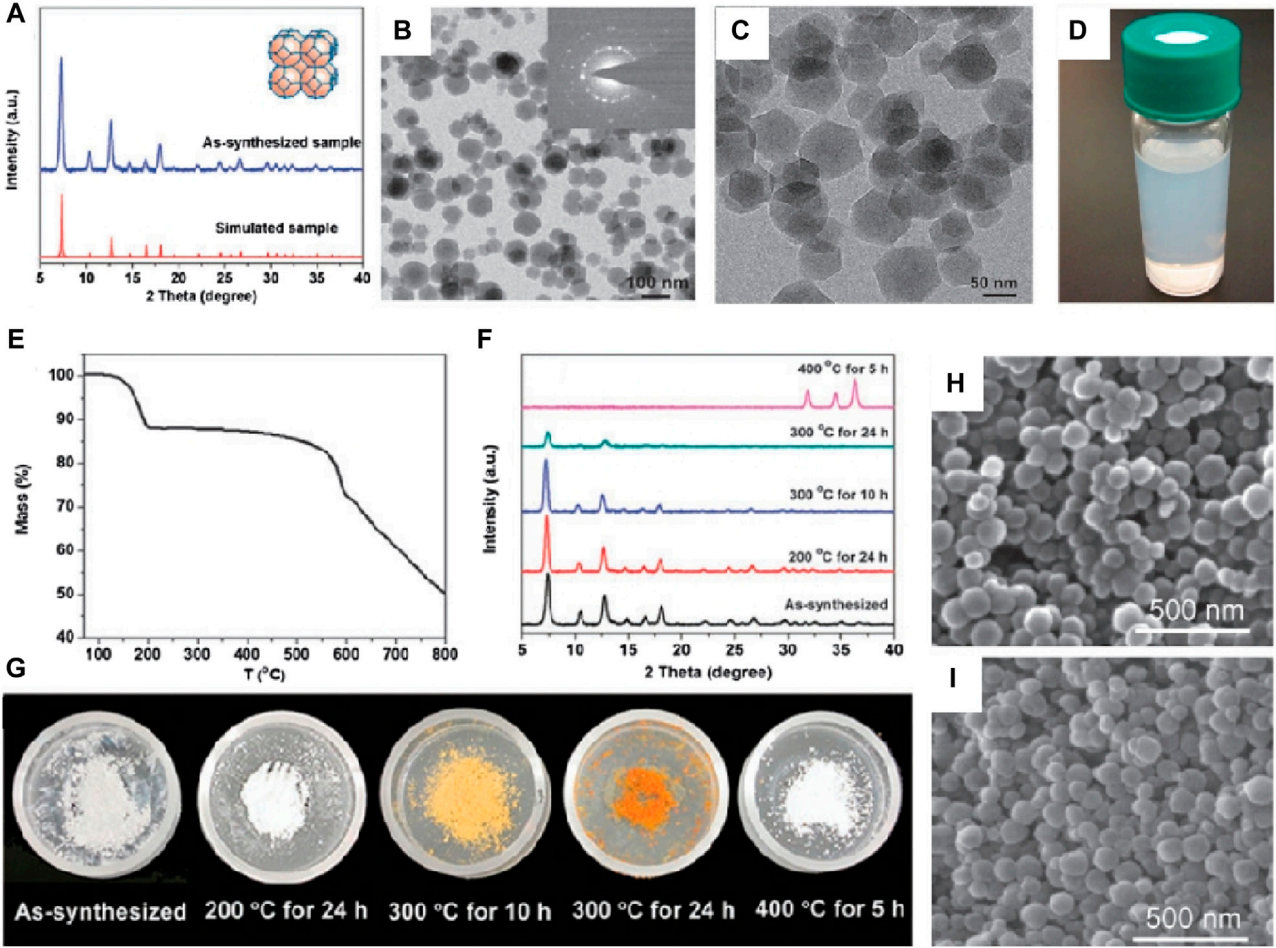
Hydrothermal synthesis of ZIF-8. **(A)** XRD patterns and **(B,C)** TME images of ZIF-8; **(D)** Electronic photograph of ZIF-8 in methanol; **(E)** TGA curve and **(F)** XRD patterns of the samples during the heating process; **(G)** Electronic photos of sample powder after heating treatment **(H,I)** SEM images of the as-synthesized sample (Pan et al., 2011).

Lai et al. firstly prepared ZIF-8 nanoparticles in aqueous solution (Pan et al., 2011), as shown in Figure 1. The transmission electron microscope images clearly revealed the hexagonal morphology of the synthesized ZIF-8 samples, with an average crystallite size of approximately 70 nm calculated using the Scherrer equation (Figures 1A, B). The X-ray diffraction patterns of the samples exhibited characteristic peaks consistent with published ZIF-8 structure data, confirming the purity of the synthesized ZIF-8 product. Thermal stability testing of the samples in air was conducted, as shown in Figures 1C–E. The color of the samples gradually changed from white to light yellow, and eventually to dark orange as the temperature increased up to 300°C (Figure 1C). However, under SEM observation (Figure 1D), the samples did not show significant changes. Instead, after heating the samples to 400°C for 5 h, their crystals transformed into smaller particles (approximately 25 nm) and the color of the samples returned to white. These results indicated the complete destruction of the ZIF-8 crystal structure and the formation of ZnO based on the XRD patterns. In summary, the hydrothermal synthesis method yielded ZIF-8 with high purity, uniform morphology, and good thermal stability.

Nowadays, this fabrication method of homogeneous, high-quality and safe ZIF-8 has become one of the important indicators of concern. Butova et al. prepared ZIF-8 with a large specific surface area (1,340 m^2^/g) via hydrothermal synthesis (Butova et al., 2017), and ZIF-8 samples exhibited high capacity in I_2_ uptake, which making it possess the ability to selectively adsorb small molecules of drugs. Munn et al. reported a method for the large-scale production of ZIF-8 (Munn et al., 2015), and the surface area of produced ZIF-8 was 1800 m^2^g^−1^. More significantly, the lab-scale and pilot-scale of the production method was reached 27 g h^−1^ and 810 g h^−1^, respectively, which greatly improved its commercialization ability.

#### 2.1.3 Microwave assisted method

The microwave-assisted method, initially introduced by Bux in 2009 (Bux et al., 2009), is an energy-efficient technology for obtaining ZIF-8. This method offers several advantages, including rapid heating rates, shorter reaction times, and higher selectivity and yield. It operates based on the interaction between electromagnetic waves and charged materials, such as polar molecules in solvents or conductive ions in solids. In comparison to the previous methods mentioned, the microwave-assisted method effectively accelerates the reaction rate and provides control over the shape and size of the ZIF-8 products. Lai et al. and Xing et al. demonstrated the synthesis of regularly rhombohedral and spherical ZIF-8 nanocrystals, respectively, using the microwave-assisted method (Lai et al., 2015; Munn et al., 2015). This method significantly reduced the reaction time compared to solvothermal synthesis. Additionally, Tang and colleagues quickly produced rhombic dodecahedron-shaped ZIF-8 nanocrystals with sizes ranging from 3 to 5 μm using microwave irradiation (Chen and Tang, 2019). The reaction time for this method was shortened to approximately 20 h. Furthermore, Bao et al. combined the advantages of the hydrothermal method with microwave irradiation to prepare ZIF-8 with a high surface area and large micropore volume. They achieved this by using a relatively low ligand to metal ion molar ratio.

For a substance to generate heat under microwave radiation, it is required to have an electric dipole (Kaufmann and Christen, 2002; De la Hoz et al., 2005). When the dipole attempts to reorient itself in response to the alternating electric field, the heating effect in microwave radiation is caused by the part that consists of an electric field, instead of the magnetic field part. This results in the generation of heat through molecular friction. During the synthesis process of ZIF-8 using the microwave-assisted method, the heat generated by dipolar polarization is primarily attributed to the interaction between polar solvent molecules like water, methanol, and ethanol (Van Tran et al., 2020). The frequency range of the oscillating field plays a crucial role in this process. It is important to determine whether the frequency range is appropriate to achieve sufficient interaction between particles. If the frequency range is excessively high, the intermolecular forces may prevent the polar molecules from moving before attempting to follow the field. This can result in inadequate inter-particle interaction. On the other hand, if the frequency range is too low, the polar molecules will have sufficient time to align themselves in phase with the field. Therefore, the microwave-assisted method is more complex compared to the previously mentioned methods, and its successful execution can be challenging in practice. It requires careful consideration of the frequency range to ensure optimal inter-particle interaction and efficient heat generation during the synthesis of ZIF-8.

#### 2.1.4 Other methods

**FIGURE 2:**
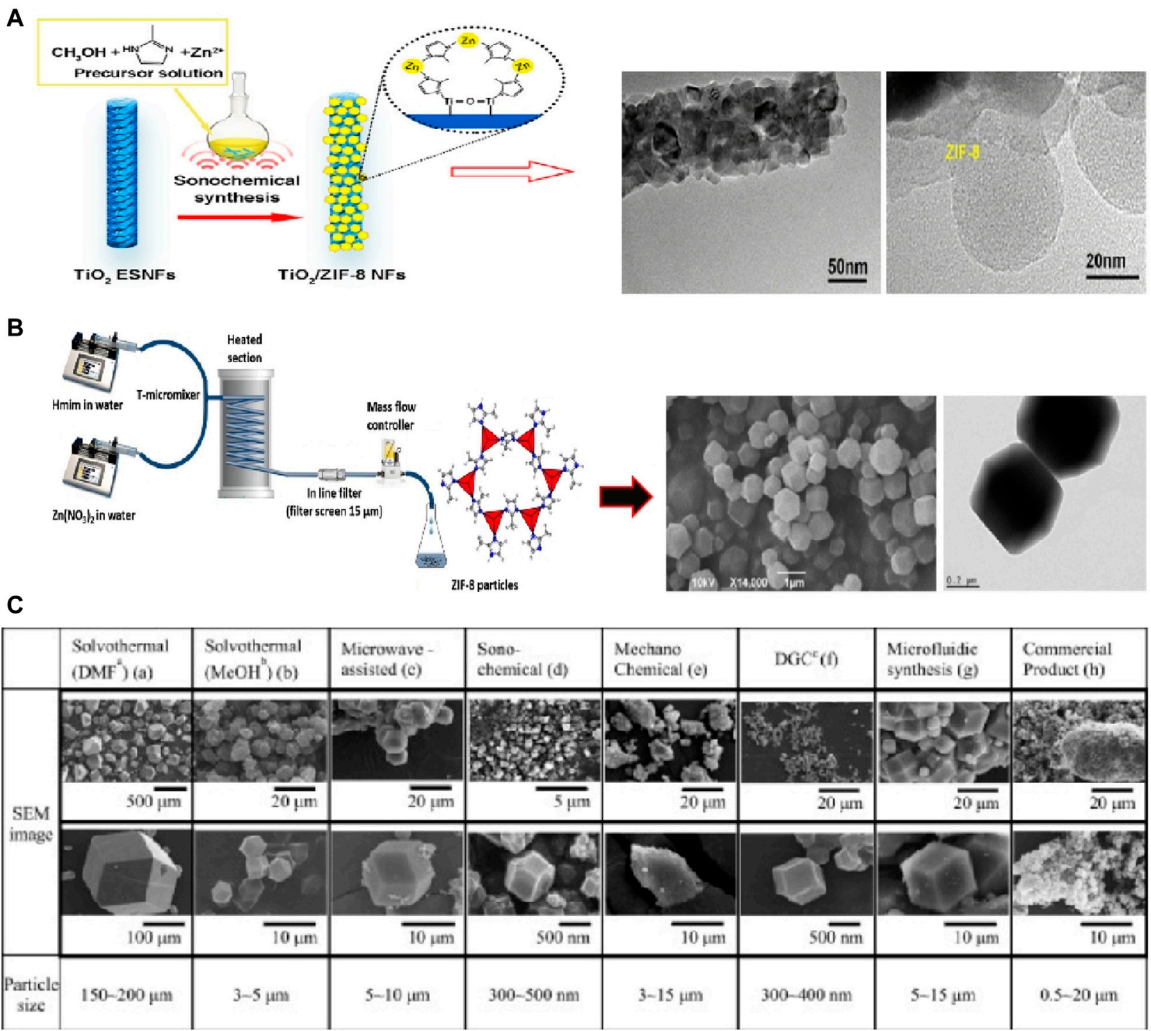
ZIF-8 prepared by **(A)** Sonochemical synthesis (Cho et al., 2013) and **(B)** mechanochemical method (Katsenis et al., 2015); **(C)** SEM images of ZIF-8 prepared via different methods (Lee et al., 2015).

In addition to solvothermal synthesis, hydrothermal method, and microwave-assisted method, there are several other synthesis routes that can be used to prepare ZIF-8 with high purity. These include sonochemical synthesis (Bui et al., 2022), mechanochemical method (Taheri et al., 2020), dry-gel conversion (Ji et al., 2020) and microfluidic method (Yao et al., 2020),. For instance, Luo et al. demonstrated the sonochemical synthesis of ZIF-8 nanoparticles on the surface of TiO2 nanofibers (Zeng et al., 2016), as depicted in Figure 2A. Ahn et al. achieved high-yield synthesis of ZIF-8 on a 1-L scale by combining sonochemical method with pH-adjusted synthesis conditions using a NaOH solution containing a small amount of triethylamine (Cho et al., 2013). Friščić et al. utilized mechanochemical methods to produce ZIF-8 particles by mixing ZnO and 2-methylimidazole and catalyzing with a small amount of acetic acid (Katsenis et al., 2015), as shown in Figure 2B. Schneider et al. employed the microfluidic method to synthesize ZIF-8 crystals with a wide size range (approximately 300–900 nm) and high specific surface area (around 1700 m^2^·g^-1^) (Kolmykov et al., 2017). These various synthesis routes offer alternative approaches for the preparation of ZIF-8 with high purity, providing flexibility and the opportunity to tailor the properties of the resulting material.

In fact, the size and morphology of ZIF-8 can vary depending on the specific preparation processes employed. For instance, Lee et al. conducted a study comparing the morphology and structure of ZIF-8 crystals using seven different synthesis methods (Lee et al., 2015), as illustrated in Figure 2C. The results revealed that while the BET surface areas of the samples fell within the range of 1,250–1,600 m^2^·g^−1^, but there were noticeable differences in the size of the crystals, ranging from several nanometers to hundreds of micrometers. Among these methods, sonochemical synthesis was capable of producing relatively homogeneous crystals. However, the unstable reaction conditions associated with this method can limit the purity of the crystals, making it challenging to achieve large-scale production. On the other hand, mechanochemical methods allow for large-scale synthesis of ZIF-8, but they may not guarantee uniformity of nanoparticles and can even result in the collapse of the crystal structure. Electrochemical methods, on the other hand, can yield ZIF-8 crystal particles with high crystallinity and relatively good properties. However, one challenge is the difficulty of separating ZIF-8 particles from the solution.

Overall, each of these techniques has its own advantages, limitations, and appropriate applications. When selecting a synthesis method for ZIF-8 based on performance requirements, it is important to consider factors such as size, shape, surface properties, strength, and thermal stability of the synthetic materials. For instance, if the goal is to investigate the effects and applications of ZIF-8 in tissue engineering, hydrothermal method may be suitable due to potential toxicity or compatibility issues. Moreover, in addition to the aforementioned approaches, researchers are actively exploring and developing new synthetic methods for the preparation of green and high-quality ZIF-8 and its derivatives. These efforts are guided by both theoretical principles and practical considerations. Examples of such emerging methods include electrochemical synthesis and ionothermal synthesis. By continuously exploring and refining synthetic routes, scientists aim to enhance the efficiency, sustainability, and control over the synthesis process, ultimately enabling the production of ZIF-8 and its derivatives with desired properties for a wide range of applications.

### 2.2 Accurate control of ZIF-8 particle size

Particle size is a crucial parameter that not only affects material performance, such as surface area and porosity, but also determines its suitability for various medical applications (Anderson et al., 2015; Hofmann-Amtenbrink et al., 2015). For instance, when it comes to drug delivery systems, the particle size used for injection should not exceed 200 nm. Additionally, particle size plays a significant role in the biodistribution of materials *in vivo*. Particles within the size range of 20–30 nm are typically absorbed and eliminated by the renal system, while particles ranging from 30 to 300 nm are absorbed by mononuclear phagocytic cells and subsequently stored in organs such as the liver, spleen, or bone marrow (as shown in Tab. 1) (Gorth et al., 2011). On the other hand, particles with sizes in the tens of microns or even millimeters range are generally unsuitable for biological applications. Over the past decade, numerous researchers have investigated the relationship between synthesis parameters and the resulting size of ZIF-8 crystals (Cao et al., 2021; Jiang X. et al., 2021). These parameters include reagent concentration, solvent, reaction time, temperature, and activators, etc (García-Palacín et al., 2020; Ghorbani et al., 2020).

**TABLE 1:** Medical applications of ZIF-8 in different sizes.

Particle size (nm)	Medical applications
<20	Surface functionalization of biomaterials
20–30	Renal system therapy
30–300	Monocyte phagocytosis
>300	Thin film

#### 2.2.1 Growth mechanism of ZIF-8 crystal

ZIF-8 belongs to the class of porous materials constructed from 2-methylimidazole coordinated to Zn^2+^. While the porous structure of ZIF-8 shares similarities with zeolites, it has distinct characteristics in terms of pore size, shape, and absorptivity. Typically, the thermal stability of MOFs is lower compared to most inorganic microporous materials, primarily due to the presence of organic linkers (Kumar et al., 2017). However, ZIF-8 stands out by exhibiting relatively high thermal stability, which may be attributed to its unique crystal growth mode. Therefore, it is crucial to investigate the growth mechanism of ZIF-8 crystals to deepen our understanding of its essence and improve control over particle size. Based on previous research, there is a consensus that the formation of ZIF-8 involves two main processes: nucleation and crystal growth.

The nucleation of ZIF-8 is primarily attributed to homogeneous nucleation, which is commonly used to explain the crystallization behavior of nanocrystalline structures formed by the connection of nitrogen or oxygen atoms to metal tetrahedral nodes (Cravillon et al., 2011). In the reaction system, the excess 2-methylimidazole undergoes protonation and then coordinates with the zinc ion, leading to the formation of a crystal nucleus (Cravillon et al., 2009). This nucleation process occurs spontaneously as it reduces the free energy of the system under conditions of concentration supersaturation. Subsequently, the growth of ZIF-8 in the solution proceeds through the diffusion and subsequent binding of small monomers on the crystal surface. Under conditions of concentration supersaturation, the nanocrystalline nucleus experiences rapid growth, resulting in the formation of ZIF-8 nanoparticles. As the solution saturation decreases, the neutral 2-methylimidazole combines with the positively charged ZIF-8, leading to the termination of the reaction. Therefore, the growth of the crystal nucleus surface is dependent on the level of reagent supersaturation.

#### 2.2.2 Precise control of ZIF-8 crystal

The regulation and control of ZIF-8 particle size can be summarized into two aspects: the regulation of reaction parameters, the introduction of surfactants and crystal regulators.

• Adjustment of reaction parameters

Adjusting reaction parameters, such as reaction time, temperature, mixing speed, and feeding ratio, is a direct and effective way to influence the characteristics of ZIF-8 powder. Choi et al. observed that the average size of ZIF-8 decreased from 155 to 35 nm as the reaction temperature increased from 120°C to 180°C (Choi et al., 2015). Similarly, Langner et al. found that the average size of ZIF-8 decreased from 78 nm to 26 nm as the synthesis temperature increased from −15°C–60°C (Tsai and Langner, 2016). These results indicate that higher reaction temperatures lead to smaller ZIF-8 grains due to increased nucleation and limited grain growth caused by the consumption of 2-methylimidazole. In addition to synthesis temperature, adjusting the charging ratio or changing the material composition can also control the particle size. Beh et al. found that increasing the concentration of Zn^2+^ enhanced the nucleation rate and suppressed nuclei growth, resulting in smaller ZIF-8 particles (Beh et al., 2018). Conversely, increasing both Zn^2+^ and 2-methylimidazole concentrations simultaneously enhanced nucleation and nuclei growth, leading to larger particle sizes. Schneider et al. investigated the effect of different zinc salts on size and morphology and found that the reactivity of the zinc salts influenced the particle size and morphology of ZIF-8 (Schejn et al., 2014). For example, the use of highly reactive zinc salts such as Zn(NO_3_)_2_, ZnSO_4_, and Zn(ClO_4_)_2_ resulted in the formation of fine ZIF-8 particles with diameters of approximately 50–200 nm. Moderately active zinc salts like ZnCl_2_ yielded particles with sizes ranging from approximately 350–650 nm. Low-reactive zinc salts led to the generation of micro-sized crystals. In fact, the reaction parameters mentioned earlier are interconnected, as the growth process of ZIF-8 involves two stages: nucleation and growth. By controlling factors such as reaction time or 2-methylimidazole concentration, it is possible to achieve smaller particle sizes at lower reaction temperatures. For instance, Beh et al. successfully prepared fine ZIF-8 particles with a mean size of approximately 60 nm by controlling the reaction time at a low temperature of 5 °C (Schejn et al., 2014). Therefore, it is worth considering the formulation of a specific combination strategy to obtain nanoparticles of the desired size.

• Introduction of surfactants agent and crystal regulator

Surfactants play a crucial role in controlling the size and morphology of nanoparticles due to their unique amphoteric molecular structure, with one end being hydrophilic and the other end being hydrophobic (Zhang and Marchant, 1996). In general, the hydrophobic end, being non-polar, inhibits crystal growth through steric effects, while the hydrophilic end with polarity often leads to high surface tension, reducing the surfactant’s adsorption capacity and affecting particle morphology (Zhu et al., 2017). As a result, surfactants can be used as blocking agents for ZIF-8 to regulate its particle size. For example, Chen et al. utilized non-ionic surfactants, specifically triblock copolymers P123 and F127, to prepare ZIF-8 particles with sizes below 104 nm and a high BET surface area of 1,599 m^2^g^−1^ under microwave irradiation (Xing et al., 2014).

Another commonly used method to control the particle size of ZIF-8 is through the use of crystallization regulators. These regulators control the nucleation process of nanoparticles by introducing crystallization inhibitors or promoters into the reaction system, thereby precisely controlling the particle size of ZIF-8. One commonly used crystallization promoter is triethylamine (TEA), which enhances the nucleation rate of ZIF-8 by accelerating the deprotonation process of 2-methylimidazole, leading to the formation of fine ZIF-8 particles. Various studies have shown that the particle size of ZIF-8 decreases with an increase in the content of TEA. On the other hand, monodentate ligands like 1-methylimidazole, formate, and n-butylamine are often used as crystallization inhibitors to assist in ZIF-8 synthesis. These ligands help adjust the proton balance during the nucleation and growth of ZIF-8 crystals. For instance, Wiebcke et al. synthesized ZIF-8 particles with a size of 10 nm by adjusting the crystal inhibitor (Cravillon et al., 2011).

### 2.3 Biocompatibility of ZIF-8

ZIF-8 has great potential in the biomedical field due to its unique structure and diverse functions. However, it is important to understand the toxicological properties of these biomaterials, as they can be influenced by factors such as chemical composition, particle size, and surface characteristics. ZIF-8 is composed of zinc clusters connected to dimethyl imidazole through coordination bonding. Zinc is an essential trace element in the human body and is involved in various physiological activities. Previous studies have shown that zinc can induce cell growth and differentiation (Ma et al., 2016). However, toxicity is a concern when zinc accumulates in the body. The threshold value for zinc ion is known to be 5 ppm (Yang Y. et al., 2020). Dimethyl imidazole, which is part of ZIF-8, is considered a safe material as the imidazole group is an important component of the amino acid group. Its content should also be controlled to ensure non-toxicity. Specifically, a concentration of 1.4 g/kg for 2-methylimidazole is considered acceptable for bio-applications (Bieniek et al., 2021).

**FIGURE 3:**
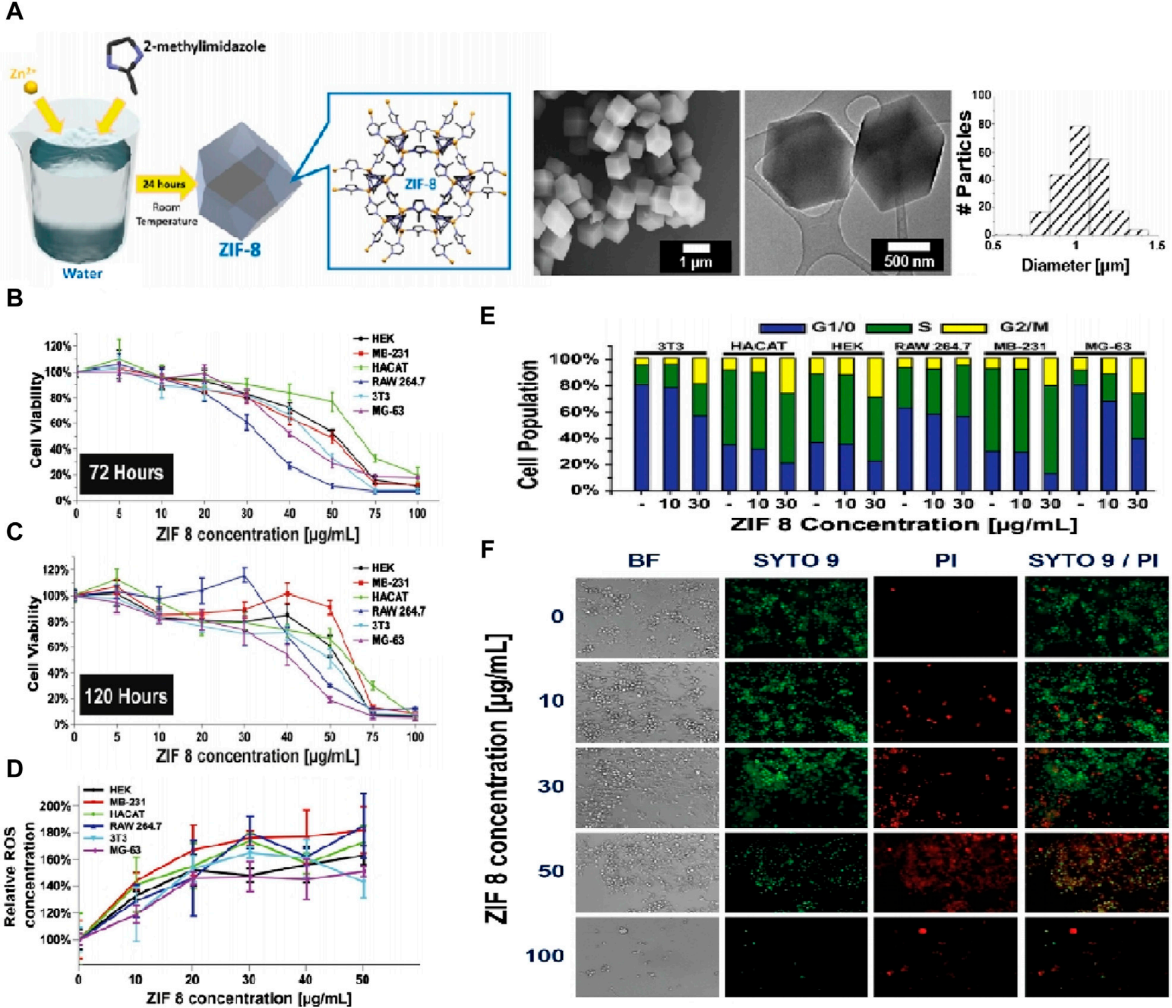
Characterization of ZIF-8 biocompatibility. **(A)** Synthesis process of ZIF-8 and corresponding TEM images; **(B,C)** MTT cell viability assay of six cell lines after 72 h and 120 h. **(D)** Calculated IC20 and IC50 values of all six cell lines; **(E)** Cell cycle analysis after treatment with different concentrations of ZIF-8 for 3 days; **(F)** Live/Dead fluorescent image of MG-63 cells cultured for different concentrations of ZIF-8 for 3 days (Hoop et al., 2018).

To evaluate the overall cytotoxicity of ZIF-8, it is important to consider the interaction between ZIF-8 components and cells. *In vitro* studies by Hoop et al. (Hoop et al., 2018) showed that ZIF-8 activated apoptosis pathways in various cell lines by affecting cell cycle arrest in the G2/M phase through the production of reactive oxygen species (ROS), as shown in Figure 3. Cell viability was significantly reduced at ZIF-8 concentrations above 30 g/mL or Zn^2+^ concentrations above 4 g/mL. *In vivo* studies by Lin et al. (Lin et al., 2021) examined the relationship between ZIF-8 concentration and tissue damage. Results showed that doses of ZIF-8 below 45 mg/kg for 24 h did not cause damage to tissues such as the heart, liver, spleen, lung, and kidney. However, doses above 45 mg/kg affected one or more platelet indices. In summary, the toxicity of ZIF-8 appears to be dependent on the injection concentration, and further studies are needed to fully evaluate its safety for *in vivo* applications.

A study by Hu et al. (Chen et al., 2020) investigated the size- and dose-dependent toxicity of ZIF-8 on human hepatoma HepG2 cells. They prepared three different-sized ZIF-8 nanoparticles (50 nm, 90 nm, and 200 nm) using the aqueous phase method and found that smaller-sized nanoparticles resulted in higher ion accumulation. Smaller nanoparticles are more easily absorbed by cells and are more likely to accumulate and remain in organs without being filtered, especially nanoparticles in the scale of 80–100 nm. Therefore, the impact of particle size on toxicity cannot be ignored when considering translational applications of nanomaterials. In addition to particle size, the surface chemistry of nanoparticles, such as hydrophobicity and surface electronegativity, also plays a crucial role in cytotoxicity. Generally, hydrophobic groups react with hydrophobic proteins on the cell membrane, causing damage to cells by altering their chemical composition. ZIF-8 nanoparticles exhibit hydrophobic properties due to their imidazole ligands, which limits their application in the biological field to some extent. To improve their biocompatibility, researchers have explored surface functionalization techniques. In crude terms, the cytotoxicity of ZIF-8 is primarily related to the injection dose. In the following sections, the versatility of ZIF-8 and its derivatives as nanoplatforms for tissue engineering applications, including drug delivery, antimicrobial activity, and cancer therapy systems, will be discussed in detail. Emphasis will be placed on highlighting *in vivo* and *in vitro* results.

## 3 Construct of multifunctional nano-platform

### 3.1 Drug delivery system

Since the development of the first sustained release formulation, Dexedrine, in the 1950s, drug delivery systems (DDS) have been recognized for their ability to safely achieve the desired therapeutic effect (Bradley, 1950). With advancements in modern technology, nanomedicine has emerged as a solution to overcome the limitations of traditional drug delivery approaches, which are often heterogeneous to both the patient and the disease. Nanoparticles, in particular, have shown great potential in improving the stability and solubility of encapsulated drugs, facilitating transport across membranes, and prolonging circulation times to enhance safety and efficacy (De Jong and Borm, 2008). Among them, MOF materials represented by ZIF-8 exhibits excellent drug delivery capabilities, and are mainly manifested in three aspects: sustained release, responsive release and programmed drug delivery.

#### 3.1.1 Sustained release system

Traditional drug treatments often face challenges related to drug specificity, a narrow window of efficacy, adverse pharmacokinetic profiles, and potential side effects (Edwards and Aronson, 2000; Huggins et al., 2012). In this regard, nanoparticle therapy aims to maximize the loading of drug molecules, thereby avoiding premature drug release and achieving more effective treatment when reaching damaged tissue sites. ZIF-8, in particular, offers advantages in this context due to its high surface area and pore volume, which enable higher drug loadings. Furthermore, ZIF-8 exhibits good degradation performance and stability in aqueous solutions, allowing for controlled and sustained release of drugs, and ultimately leading to long-lasting therapeutic effects.

In tissue regeneration treatments, the required drug functions typically include antibacterial, anti-inflammatory, and regeneration-promoting properties. To prevent premature drug leakage before reaching the target location, the most effective approach is to encapsulate drugs during the synthesis process of ZIF-8. Zhu et al. utilized ZIF-8 to encapsulate biological cascade enzymes and combined them with antisense oligonucleotides (ASOs) to create a biomineralized nanomaterial (GOx&HRP@ZIF-8/ASO), aiming to achieve efficient antibacterial effects (Zhang Y. et al., 2022). The results showed that the composites exhibited excellent antibacterial properties, with a concentration of only 16 μg/mL for MRSA bacteria after treatment. Additionally, the composites demonstrated superior biofilm destruction ability, with a bacteria removal efficiency of 88.2%, compared to simple ZIF-8 (32.85%) and ftsZ ASO (58.65%). This indicates that the production of reactive oxygen species (ROS) by biological cascade enzymes was fully utilized.

In a study conducted by Wang et al. (Zhang et al., 2023), dimethyloxallyl glycine (DMOG) was loaded into the ZIF-8 skeleton to create a drug-loading system aimed at promoting osteogenesis-angiogenesis coupling. Transmission electron microscopy (TEM) images revealed that the DMOG@ZIF-8 nanoparticles exhibited a crystal morphology similar to ZIF-8. X-ray photoelectron spectroscopy (XPS) analysis indicated that the coupling between DMOG and ZIF-8 was achieved through the binding of C=O and C-O bonds.

**FIGURE 4:**
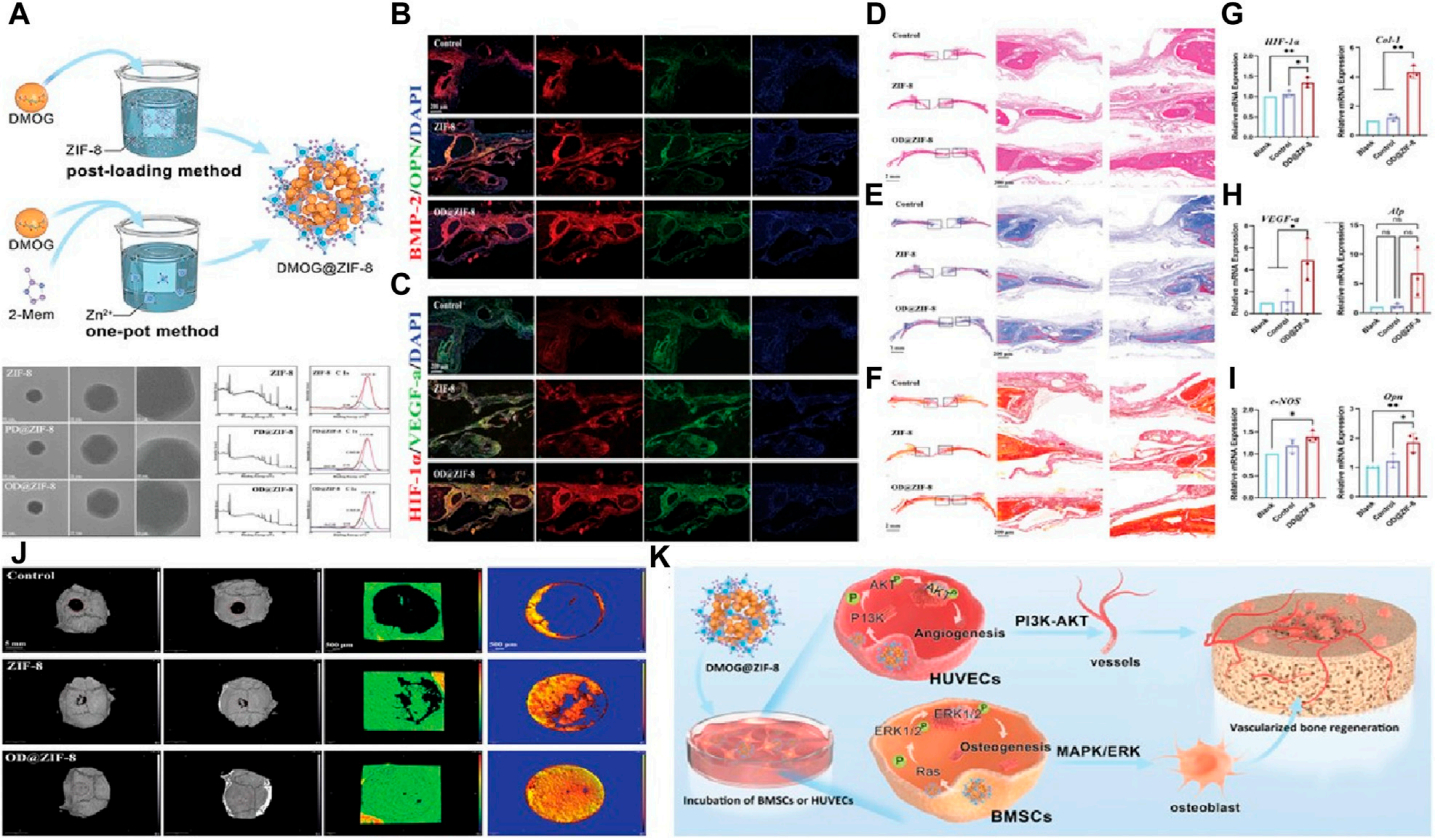
Construction of a sustained drug release system through ZIF-8 **(A)** The synthesis mechanism of nanoparticles and their TEM images and XPS results; Fluorescence images by **(B)** BMP-2 and OPN staining and **(C)** HIF-1α and VEGF-a staining; Images of bone formation in the defect area via **(D)** H&E, **(E)** Masson’strichrome and **(F)** Sirius Red staining; mRNA expression using RT-PCR analysis **(J)** Experimental schematic of rat skull repair; RT-PCR analysis of the mRNA expression including **(G)** HIF-1α and Col-1, **(H)** VEGF-a and ALP, **(I)** eNOS and Opn; **(K)** Osteogenic mechanism via using DMOG@ZIF-8 (Zhang et al., 2023).

To assess the vascularization and bone regeneration effects of DMOG@ZIF-8 nanoparticles, the authors prepared implants containing DMOG@ZIF-8 using sodium alginate (Figure 4A). They further studied the osteogenic activity of DMOG@ZIF-8 by utilizing a rat defect model. Immunofluorescence staining was performed to detect signal transduction related to angiogenesis and osteogenesis during bone regeneration (Figures 4B, C). The results showed that the DMOG@ZIF-8 group exhibited higher intensity of BMP-2, OPN, OCN, CD31, HIF-1α, and VEGF-a compared to the other groups. This indicates that DMOG@ZIF-8 effectively regulated angiogenesis and promoted osteogenic ability. Furthermore, the newly formed bone was investigated (Figures 4D–J). It was evident that DMOG@ZIF-8 effectively promoted new bone formation, and the high expression of related osteogenic and angiogenic genes further confirmed this observation. In summary, DMOG@ZIF-8 demonstrated enhanced migration of human umbilical vein endothelial cells, secretion of angiogenesis-related proteins, and extracellular matrix mineralization, thereby promoting vascularized bone formation after implantation (Figure 4K).

For tissue defect produced by bone tumor, it was expected that nanoparticles have anti-tumor functions apart from the aforementioned functions. In this context, Luan et al. utilized ZIF-8 to absorb indocyanine green (IR820), and then functionalized with hyaluronic acid (hydroxyapatite) to composite nanoparticles with targeted functions (Zhang et al., 2018). Results showed the nanoparticles could effectively target tumors and inactivate tumor cells. Fang et al. used ZIF-8 nanoparticles to encapsulate autophagy inhibitor 3-methyladenine (3-MA) to slow-control its release before reaching the target (Chen et al., 2018). Cell tests revealed that 3-MA@ZIF-8 exhibited excellent antitumor efficacy and effectively inhibited the expression of autophagy-related markers (Beclin 1 and LC3).

#### 3.1.2 pH-responsive delivery system

The application of stimuli-responsive nanocarriers in drug delivery has become an interesting opportunity for optimizing tissue regeneration therapy (Mura et al., 2013). In a stimuli-responsive delivery system, the drugs loaded into nanoparticles can be released in response to specific stimuli such as pH, glucose, light, or temperature (Ganta et al., 2008). This allows the nanocarriers to specifically respond to pathological triggers present at selected target sites, making them a promising approach for tissue engineering. Among the various stimuli-responsive properties, pH responsiveness has been particularly considered as promising for tissue engineering, as both the pH at defect sites and in tumor environments are lower than that of normal tissue (Zhu and Chen, 2015; Qian et al., 2023a). By designing nanocarriers with pH-responsive properties, drug release can be specifically triggered in damaged tissue rather than normal tissue.

In a pH-responsive delivery system, ZIF-8 offers inherent advantages compared to stimuli-responsive nanocarriers synthesized through complex processes. ZIF-8 is relatively stable in aqueous solutions and its degradation rate increases as the environmental pH decreases, making it more suitable for treating tissue damage. Tyagi et al. (Kaur et al., 2017) utilized ZIF-8 to load 6-mercaptopurine (6-MP) and found that 6-MP@ZIF-8 exhibited significantly faster drug release in acidic pH compared to pH 7.4. Gai et al. (Sun et al., 2019) designed functional core-shell nanoparticles by encapsulating unstable d-α-Tocopherol succinate (α-TOS) within ZIF-8 and coating it with hyaluronic acid (hydroxyapatite). The results showed that the HA shell served as a tumor-targeted “guider,” connecting with tissue tumors through the CD44-mediated pathway. Subsequently, the decomposition of the ZIF-8 core released the loaded α-TOS in the acidic microenvironment of the tumor.

#### 3.1.3 Programmable delivery systems

Tissue repair is a highly intricate process that requires coordinated events to ensure successful healing. In conditions like osteomyelitis, incomplete removal of bacterial infection at the defect site can lead to necrosis of newly formed bone tissue (Li et al., 2016). Therefore, it becomes crucial to develop a programmable delivery system that offers precise control over the timing and dosage of drug release in the patient’s body. Ideally, nanoparticles with programmable delivery functionality can improve therapeutic effectiveness by allowing control over the timing, duration, dose, and site-specific release of drugs in a predictable, repeatable, and on-demand manner. This programmability enables enhanced treatment outcomes and personalized medicine approaches.

A straightforward and effective method to encapsulate drugs in ZIF-8 is through chemical binding or physical adsorption. By utilizing this approach, controlled release of drugs can be achieved in a spatial and temporal manner, either by surface functionalizing ZIF-8 or by designing another functional material using ZIF-8 as a precursor. Ren et al. (Li et al., 2016) developed anti-tumor core-shell nanoparticles called ZDOS NPs, which had a dual function. The ZIF-8 core of the nanoparticles was loaded with DOX (doxorubicin), while the shell consisted of silicon-containing disulfide bonds. Under physiological conditions, ZDOS NPs remained stable. However, in the presence of endogenous glutathione in tumor cells, the disulfide bonds were ruptured, exposing the ZIF-8 core. This triggered the controlled release of DOX, enabling programmed drug delivery. Wang et al. (Tan et al., 2022) embedded silver nanoparticles and emodin (Phy) into the structure of ZIF-8 and modified it with hyaluronic acid (hydroxyapatite) to create composite particles with synergistic antimicrobial activity. The HA component of the nanoparticles underwent decomposition due to the secretion of hyaluronidase by bacterial growth, causing its accumulation around the bacteria. As the bacteria metabolized, the microenvironment underwent slight acidification, triggering the pH-responsive release of Phy and Ag.

**FIGURE 5:**
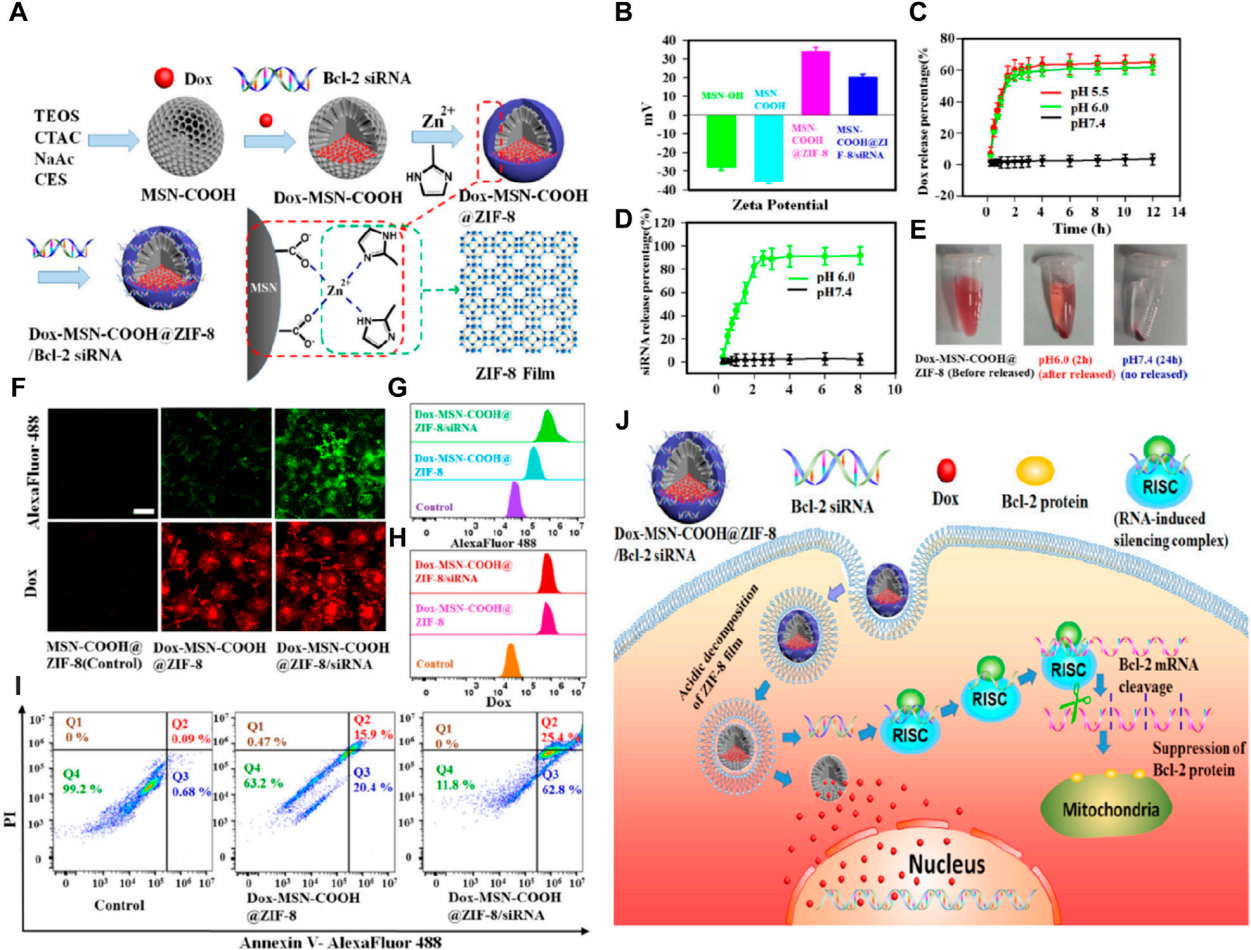
Construction of a programmable delivery system through ZIF-8. **(A)** Synthesis process of the nanoparticles; **(B)** Zeta potential of the samples; **(C)** Release situation of Dox in nanoparticles at different pH values; **(D)** Release situation of *in vitro* FAM-labeled siRNA for the nanoparticles; **(E)** Electronic photos of the nanoparticles dispersed in PBS with pH 6.0 (2 h) and pH 7.4 (24 h); **(F)** CLSM images of MCF-7/ADR cells and **(G)** corresponding flow cytometry analysis after incubation with the nanoparticles for 72 h **(H)** Annexin V/PI analysis of MCF-7/ADR cells after the incubation with the samples; **(I)** Antitumor mechanism of samples (Pan et al., 2018).

Chu et al. (Pan et al., 2018) developed a novel drug delivery system where a nanometer-thick ZIF-8 film was grown *in situ* on the surface of carboxylated mesoporous silica (MSN-COOH) nanoparticles, as illustrated in Figure 5. In this design, the ZIF-8 membrane facilitated the transformation of the charge of MSN-COOH from negative to positive through electrostatic interactions, enabling effective loading of siRNA (small interfering RNA). The positively charged membrane enhanced cellular uptake of the nanoparticles and promoted their escape from lysosomes. Moreover, the ZIF-8 membrane decomposed in the acidic lysosomal environment, leading to the intracellular release of siRNA. Upon entering the cell, the ultra-thin ZIF-8 membrane would decompose in acidic lysosomes, triggering the release of both siRNA and drugs. These experiments demonstrated that the nanoparticles significantly enhanced the therapeutic effect on cancer cells with multiple drug resistance.

### 3.2 Phototherapy system

Phototherapy, which encompasses photodynamic therapy (PDT) and photothermal therapy (PTT), is emerging as an alternative option to traditional drug therapies in tissue engineering (Liu S. et al., 2020; Li et al., 2020). PDT is a minimally invasive therapeutic approach that utilizes a photosensitizing agent and specific wavelengths of light to selectively destroy abnormal cells and bacteria (Chiu et al., 2005). Through the activation of photosensitizers, PDT generates ROS by reacting with ambient oxygen sources, leading to the destruction of abnormal cells and bacteria. On the other hand, PTT achieves cell death by inducing thermal damage using an external light source, typically near infrared (near-infrared) light, and a photothermal agent (Bai et al., 2019). Furthermore, PTT has the additional advantage of generating heat, which can be utilized to promote tissue growth and regeneration. For instance, PTT can stimulate the growth of blood vessels in ischemic tissues and facilitate bone tissue growth in fractures and cases of osteoporosis (Paul et al., 2014). However, the therapeutic efficacy of a single modality is limited in the context of tissue repair. Therefore, combining PTT or PDT with other treatment methods such as chemodynamic therapy (CDT) is crucial to achieve superior therapeutic outcomes. In this regard, the surface functionalization characteristics of ZIF-8 are instrumental in constructing a nanoplatform for multimodal collaborative therapy. This section provides a detailed summary of three multimodal collaborative therapies involving ZIF-8, namely, chemical photothermal therapy, chemical photodynamic therapy, and photothermal photodynamic therapy.

#### 3.2.1 Synergistic chemo-photothermal therapy

Recently, many studies have been conducted exploring ZIF-8 for its potential in drug delivery. However, conventional drug therapies often face challenges in clinical practice, such as uncontrolled release and drug resistance. To address these issues, the combination of photothermal therapy (PTT) and chemotherapy has emerged as a promising approach for achieving more efficient therapeutic effects in complex tissue repair scenarios. For instance, Yin et al. developed a targeted drug delivery system for osteosarcoma using polydopamine (pDA)-modified ZIF-8 nanoparticles loaded with methotrexate (MTX) (Yin et al., 2022). In this system, ZIF-8 served as a stable platform for drug loading and targeted delivery, while pDA modification prevented excessive drug release and imparted excellent photothermal effects and biocompatibility to the delivery system. Biological assays demonstrated that the pDA/MTX@ZIF-8 nanoparticles induced apoptotic cell death in MG63 cells through the controlled release of MTX. Moreover, the introduction of PTT effectively enhanced the anti-tumor effects and reduced the dosage of chemotherapy drugs. The results showcased the synergistic chemo-photothermal therapy effect of pDA/MTX@ZIF-8 nanoparticles (combination index CI = 0.346) and their exceptional biocompatibility, highlighting their potential for osteosarcoma therapy.

**FIGURE 6:**
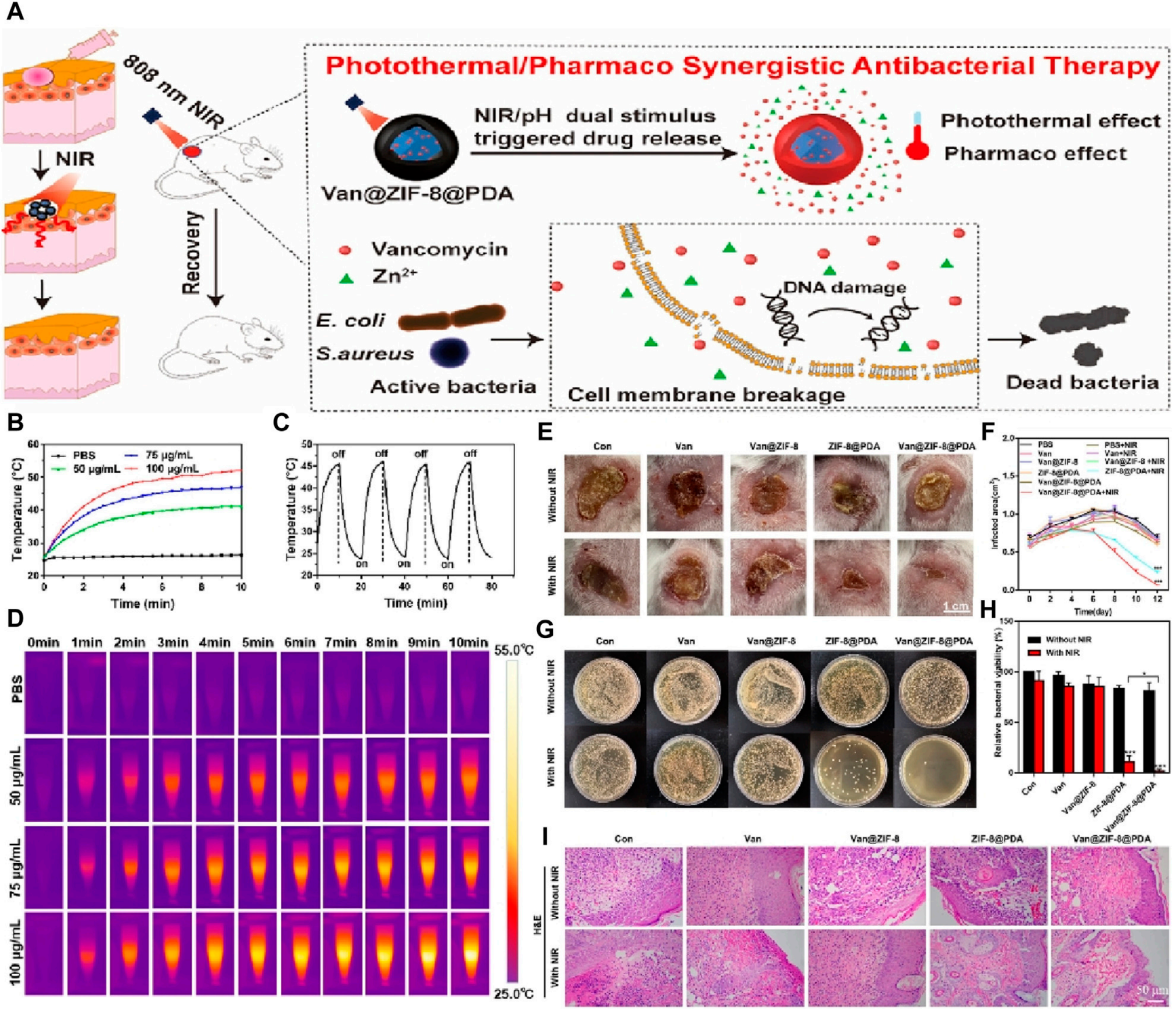
**(A)** Schematic diagram for the efficacy of a dual spike responsive MOF based nanosystem; **(B)** Photothermal effect test of different nanoparticle concentrations in PBS under NIR irradiation (1.0 W/cm2) over 10 min; **(C)** Photothermal stability test of the nanoparticle with concentration of 75 μg/mL; **(D)** Photothermal imaging images of different nanoparticle concentrations; **(E)** Digital photos of infected skin in mice treated with different nanoparticle concentrations for 12 days (PBS, Van: 3.62 μg/mL, Van@ZIF-8: 53.95 μg/mL, ZIF-8@PDA: 96.38 μg/mL, and Van@ZIF-8@PDA: 100 μg/mL); **(F)** Quantification of the area of the infected skin of mice from each treatment group over time; **(G)** Colony photos and **(H)** corresponding bacterial activity analysis obtained from infected skin tissue of each treatment group; **(I)** H&E staining images of infected skin tissue from each treatment group at days 12 (Xiao et al., 2021).

Another noteworthy study by Xiao et al. focused on the development of antibacterial agents with dual stimuli-responsive capabilities using ZIF-8 as a binder (Xiao et al., 2021). The researchers encapsulated vancomycin within ZIF-8, which was surface-modified with polydopamine. This approach enabled the combined effect of near-infrared (near-infrared) light-activated hyperthermia and the pH-responsive properties of ZIF-8. As a result, the system achieved efficient drug delivery, effectively eliminating planktonic bacteria and preventing biofilm formation, as shown in Figure 6. Furthermore, cell testing demonstrated that Van@ZIF-8@PDA exhibited minimal toxicity, thereby reducing the required antibiotic concentrations for bacterial eradication. The researchers also verified the excellent biocompatibility of the system *in vivo* using a mouse skin abscess model infected with Mu50. Overall, this work presents a promising strategy for combating antibiotic-resistant bacterial infections.

As well known, near-infrared light is commonly divided into two biologically transparent windows: the near-infrared I window (650–950 nm) and the near-infrared II window (1,000–1,350 nm) (Tsai et al., 2013). The near-infrared II window offers deeper tissue penetration due to reduced absorption and scattering effects. Therefore, utilizing the photothermal effect within the near-infrared II window is particularly meaningful for deep tissue repair. Deng et al. developed a yolk-shell structured drug carrier, Au@MOF, with remote-controlled and stimuli-responsive functions. The carrier consisted of star-shaped gold (Au star) as the photothermal yolk and biodegradable ZIF-8 as the shell (Deng et al., 2019). The chemotherapeutic drug, doxorubicin hydrochloride (DOX), was encapsulated within the cavity, enabling controlled release behavior through the degradation process of ZIF-8 in the mildly acidic tumor microenvironment. Upon 1,064 nm laser irradiation, the gold nanostar@ZIF-8 system effectively killed tumor cells through the synergistic effects of photothermal therapy and triggered drug release. Additionally, the strong absorbance of the system in the NIR region allowed for thermal imaging and photoacoustic imaging capabilities.

#### 3.2.2 Synergistic chemo-photodynamic therapy

**FIGURE 7:**
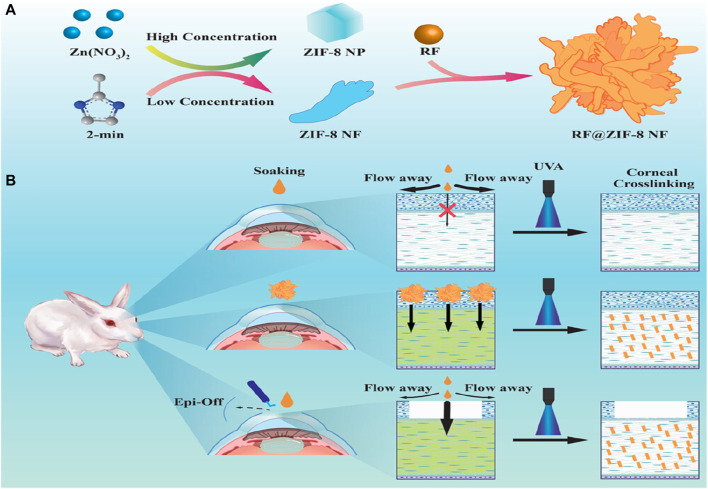
The **(A)** synthesis process of RF@ZIF-8 and **(B)** the mechanism of CXL treatment (Yang M. et al., 2022).

PDT is a non-invasive treatment where photosensitizers absorb energy from a specific light source, transferring it to surrounding oxygen molecules to generate ROS and eliminate abnormal cells. PDT combined with drug therapy offers a promising approach for addressing tissue repair issues caused by tumors or bacterial infections. Chen et al. developed a novel drug delivery system based on ZIF-8 for synergistic chemotherapy and PDT in endophthalmitis treatment (Chen et al., 2019). The system involved the *in situ* growth of silver (Ag) and subsequent modification with vancomycin/NH_2_-polyethylene glycol (Van/NH_2_-PEG) on the surface of ZIF-8 loaded with the photosensitizer ammonium methylbenzene blue (MB). *In vitro* bacterial tests demonstrated high efficacy in chemotherapy and photodynamic therapy against *Staphylococcus aureus*, *Escherichia coli*, and MRSA. Moreover, an *in vivo* mouse endophthalmitis model confirmed the biocompatibility and antibacterial properties of the composite nanomaterials. The study’s phototherapy synergistic chemotherapy approach, taking advantage of the inherent transparency of the eyes, holds promising potential for ophthalmic disease applications. Additionally, Yang et al. utilized ZIF-8 as a carrier to prepare hibiscus-like nanoparticles (6RF@ZIF-8) for the treatment of corneal cross-linking (CXL) using the common photosensitizer riboflavin-5-phosphate (RF) (Yang M. et al., 2022). The results demonstrated good compatibility and improved CXL effects compared to conventional protocols, with the added benefit of relieving postoperative eye pain and reducing the risk of infectious keratitis (Figure 7).

#### 3.2.3 Synergistic chemotherapy, PTT and PDT

Considering the superiority of phototherapy, combining PTT and PDT in an appropriate way may bring desirable treatment outcomes. Tian et al. developed hybrid nanoparticles consisting of a porphyrin-based organic polymer (POP) coated on ZIF-8 (POP@ZIF-8) for cancer photodynamic and photothermal therapy (Tian et al., 2023). The pH-responsive properties of ZIF-8 enable the accurate delivery of the photosensitizer (porphyrin-based POP) to tumor areas, where it can effectively deactivate abnormal tumor cells through PDT. Simultaneously, the composite materials can convert light into local high heat, causing damage to tumor cells under laser irradiation and synergistically promoting the production of reactive oxygen species, thus enhancing the effectiveness of PDT. In cases where persistent pathogen infections delay the wound healing process and bring uncertain complications, Zhang et al. coencapsulated the phytochemical curcumin (Cur) and indocyanine green (ICG) into ZIF-8 for PDT/PDT sterilization (Zhang S. et al., 2022). The results demonstrated that the composite material exhibited efficient and rapid antibacterial performance through the synergistic effect of photothermal and photodynamic therapy, achieving a bactericidal rate of over 99%. Additionally, the material showed good compatibility, contributing to the rapid recovery of infected wound sites with minimal biological burden.

## 4 ZIF-8 based tissue engineering scaffold

Tissue engineering, a branch of regenerative medical technology, focuses on therapeutic strategies and tissue regeneration. It utilizes bio-polymeric scaffolds combined with functional materials or growth factors to facilitate tissue regeneration. In this context, bio-polymeric scaffolds should possess mechanical or degradable properties similar to those of the implanted tissues, while functional nanoparticles need to exhibit specific therapeutic functions within the body. As discussed earlier, ZIF-8 demonstrates great potential in constructing functional nanoparticles. The following sections provide a detailed discussion on how ZIF-8 can be utilized for repairing damaged cells in tissue engineering, specifically focusing on bone, nerve skin, and vascular tissue.

### 4.1 Bone tissue scaffold

Bone tissue engineering plays a crucial role in tissue regeneration by aiming to develop biomaterials that facilitate osteoblast proliferation and support natural bone mineralization processes (Salgado et al., 2004). Loss or dysfunction of bone tissue due to trauma, injury, disease, or aging can lead to significant morbidity and various socio-economic issues (Feng et al., 2023b). Due to limitations in the availability of autologous tissues and the risk of immune rejection with allogeneic tissues, artificial tissue transplantation has become the primary approach for bone tissue repair (Gao et al., 2023). It is well-known that the repair and regeneration of bone tissue is a complex and time-consuming process, typically taking around 8–12 months (Zan et al., 2022). Therefore, an ideal artificial bone scaffold should possess suitable biomechanical properties and degradation rate, while also promoting bone tissue regeneration.

Biodegradable polymers such as PGA, PLLA, and PLGA have emerged as promising matrix materials for bone repair in tissue engineering due to their wide availability, non-immune rejection, and natural degradability. However, these materials face challenges regarding their mechanical properties and biological activity. In recent studies, ZIF-8 and its derivatives have shown potential in bone tissue engineering. When incorporated into polymer scaffolds, ZIF-8 acts as a nano reinforcing phase, enhancing the mechanical properties of the scaffolds. Its hydrophobic functional groups enable good interfacial bonding with most polymers (Zhang et al., 2012). Shuai et al. utilized selective laser sintering to fabricate a PLLA/ZIF-8 scaffold (Yang Y. et al., 2020). Mechanical tests demonstrated that the incorporation of 2 wt% ZIF-8 increased tensile strength by 36.9% and compressive strength by 85.6%. The amino groups of ZIF-8 formed hydrogen bonds with the carboxyl groups of PLLA, improving the interface bonding and preventing nanoparticle aggregation. Importantly, the introduction of ZIF-8 in the PLLA scaffold accelerated the degradation rate of PLLA due to its responsive dissolution characteristic, creating an autonomous cycle process.

**FIGURE 8:**
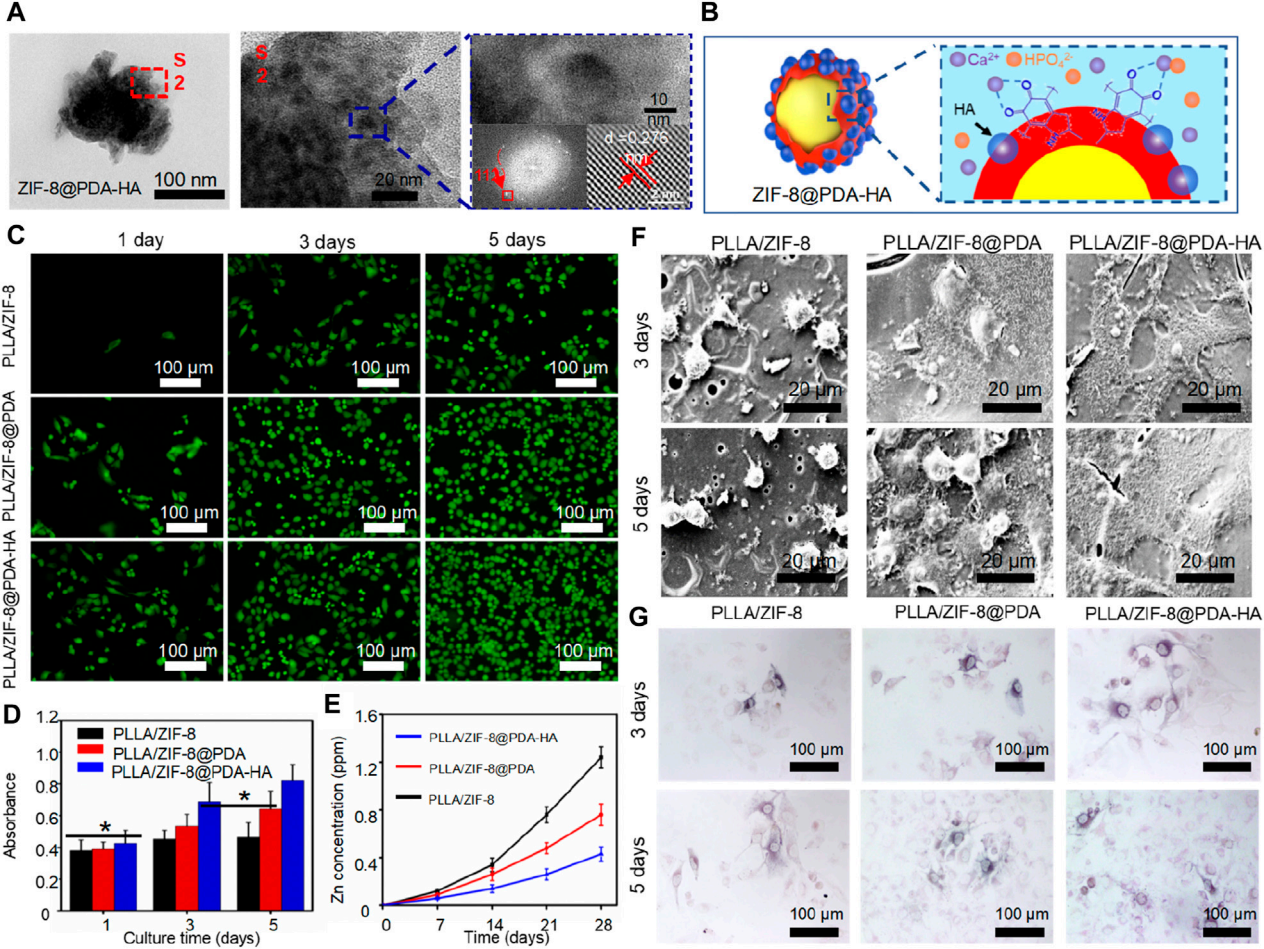
**(A)** TEM images of ZIF-8@PDA-HA nanoparticles and **(B)** its synthesis mechanism; **(C)** Live/death cell fluorescence images and **(D)** CCK-8 results of MG-63 cells cultured on the scaffold; **(E)** Release concentration of Zn^2+^ in PBS; **(F)** Cell adhesion and **(G)** ALP expression of MG-63 cells cultured on the scaffold (Shuai et al., 2021).

Apart from improving the physicochemical properties of polymer scaffolds, ZIF-8 itself has the ability to promote bone tissue growth through the controlled release of zinc ions. Zinc ions have been shown to have a positive impact on tissue regeneration. Previous research has demonstrated that a PLLA/ZIF-8 scaffold can induce cell growth and differentiation, outperforming pure PLLA scaffolds (Yang Y. et al., 2020). Furthermore, Shuai et al. developed a core-shell structured nanoparticle by inducing the *in situ* growth of hydroxyapatite (hydroxyapatite) on ZIF-8 nanoparticles using polydopamine (PDA) (Figure 8). ZIF-8 served as the core, while hydroxyapatite (hydroxyapatite) formed the shell (Shuai et al., 2021). This core-shell structured nanoparticle, known as ZIF-8@PDA-HA, was then incorporated into the PLLA scaffold for bone tissue engineering purposes.

Hydroxyapatite (HA) is well-known for its excellent osteogenic activity, which imparts mineralization ability to the scaffold and works in collaboration with ZIF-8 to promote bone tissue regeneration. Furthermore, the *in situ* synthesis of HA and polydopamine (PDA) creates a shielding layer that controls the release of Zn^2+^ ions, preventing cytotoxicity caused by excessive metal ions. Experimental results demonstrated that the PLLA/ZIF-8@PDA-HA scaffold exhibited superior bioactivity and osteogenic performance. After 28 days of immersion, the Zn2+ concentration in the PLLA/ZIF-8@PDA-HA scaffold decreased by 65.3%. By utilizing the pZIF-8 drug carrier, the scaffold enabled spatial and temporal release patterns of BMP-2 and cisplatin, contributing to effective bone formation. Cell tests confirmed that the scaffold accelerated the rate of new bone formation.

**FIGURE 9:**
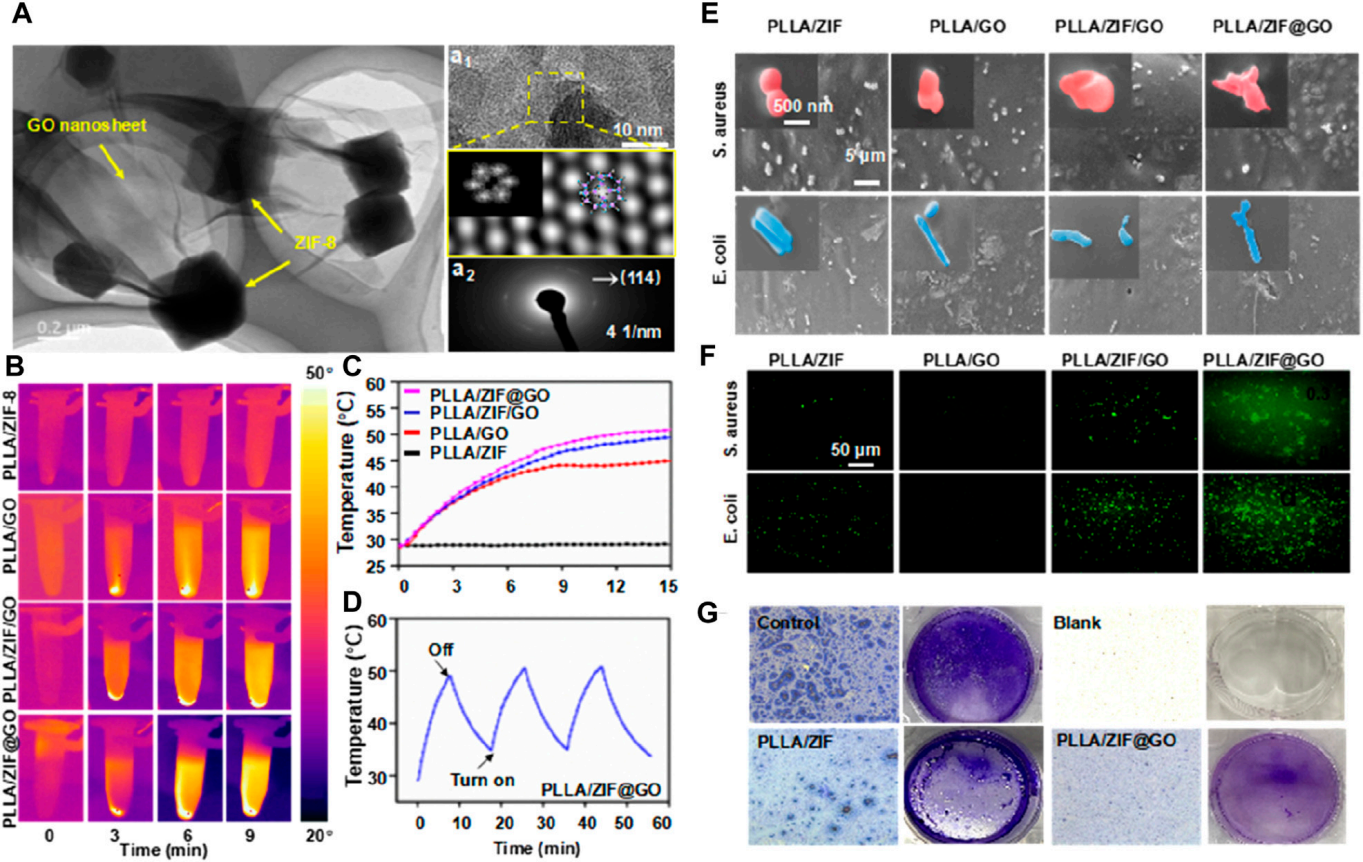
**(A)** TEM images of ZIF-8@GO (a1) high-resolutionTEM, (a2) SADPs, and (d3) EDS map of ZIF-8@GO; **(B)** Thermal photos and **(C)** temperature curves of immersion solution for all scaffold groups; **(D)** Photothermal stability test via recycling−heating profiles; **(E)** SEM images of bacterial morphology on the scaffold; **(F)** Fluorescence images to detect ROS production; **(G)** Light microscopy images of the samples treated with crystal violet (Yang Y. et al., 2022).

Patients with bone defects commonly receive injections of antibiotics and anti-inflammatory drugs to prevent bacterial infections and associated inflammatory reactions (Qi et al., 2023a; Qian et al., 2023b). Previous studies have indicated that Zn^2+^ ions released by ZIF-8 possess antibacterial properties and certain anti-inflammatory effects (Mutlu et al., 2022). Therefore, incorporating ZIF-8 into a biopolymer scaffold can further enhance the quality of implants (Mutlu et al., 2022). To enhance the antibacterial efficacy of bone scaffolds, Yang et al. developed a chemo-photothermal collaborative nanosystem called ZIF-8@GO (Yang Y. et al., 2022), as depicted in Figure 9. In this system, the photothermal effect of graphene oxide (GO) generated a temperature of approximately 50 °C, enhancing the permeability of bacterial biofilms. Concurrently, Zn^2+^ ions were more likely to enter bacterial cell membranes and inactivate the bacteria, resulting in efficient sterilization. ZIF-8@GO was then incorporated into the PLLA scaffold. Antibacterial tests demonstrated that the composite scaffold exhibited excellent antibacterial properties, with an antibacterial rate of 85% and enhanced cell proliferation.

### 4.2 Nerve tissue scaffolds

When tissue injuries occur due to accidents or diseases, nerve damage or loss often accompanies them, given the widespread distribution of peripheral nerves throughout the body (Towbin, 1970; Qi et al., 2023b). However, the shortage of autologous nerve donors poses a significant clinical challenge for the regeneration and functional recovery of peripheral nerves. In this context, nerve guidance conduits (NGCs) have emerged as a promising alternative to autologous transplantation in the field of neural tissue engineering. The key requirements for nervous system repair include promoting axonal growth and supporting long-distance regeneration (Olson, 1997). The drug-loading capabilities of ZIF-8 can be tailored to meet the specific needs of nerve regeneration. Additionally, ZIF-8 exhibits desirable characteristics for nerve tissue repair, such as the ability of released Zn^2+^ ions to participate in the regulation of information transmission and synaptic plasticity between neurons (Smart et al., 2004).

MicroRNAs (miRNAs) are noncoding RNAs that are naturally expressed within cells. They possess the ability to regulate cellular behavior, exhibit stable activity, and can be easily synthesized (Beermann et al., 2016). Recent research has demonstrated the potential application value of miRNAs in promoting axonal elongation of PC12 cells by modulating the PI3K/Akt/mTOR pathway, highlighting their significance in nerve tissue engineering (Qi et al., 2023b). However, exposed miRNAs face challenges in entering cells on their own. To address this issue, Wang et al. employed ZIF-8 nanoparticles to load miRNA-29 and incorporated them into a SF/GT hydrogel catheter. This approach aimed to enhance the biological activity and neural repair effects of miRNA-29a by enabling its sustained release (Wang et al., 2023). *In vitro* cell tests showed that the composite conduit significantly facilitated Schwann cell myelination, neuronal differentiation, and axon extension in PC12 cells. Furthermore, the conduit regulated the inflammatory microenvironment of nerve regeneration, contributing to its overall effectiveness.

**FIGURE 10:**
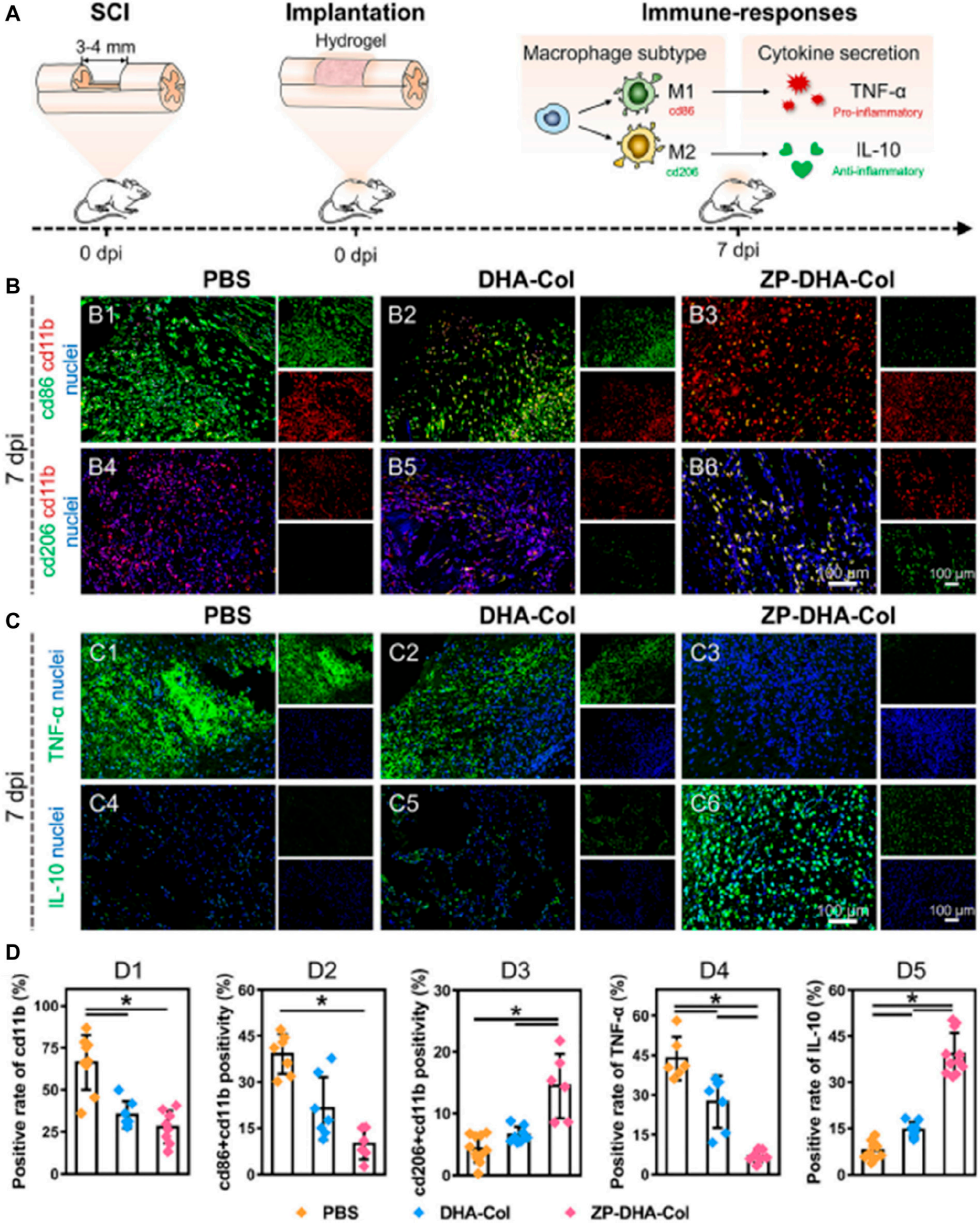
Neuroprotective effect of the microfibers *in vivo* (Xin et al., 2022) **(A)** Schematic diagram of the immune response of the sample; **(B,C)** Represent ative immunofluorescence images of spinal tissue; **(D)** Quantitative data of cd11b positivity (D1), cd86 + cd11b double positivity (D2), cd206 + cd11b double positivity (D3), TNF-α positivity (D4), and IL-10 positivity (D5) per visual field.

Injuries to the spinal cord represent a common situation in neural tissue engineering, where interference with communication among intact neurons leads to deterioration of neurons and cellular demise. Therefore, building a strong connection between injured nerves is crucial for achieving restoration of both structure and function in cases of Spinal cord injury (Guéz et al., 2003). It is important to note that nerve repair not only involves axonal regeneration but also necessitates the prevention of excessive inflammation. Damaged cells release “risk factors” and recruit immune cells, triggering an inflammatory response. If not properly managed, this response can lead to secondary damage and disease progression. In their study, Xin et al. successfully loaded interleukin 4 (IL-4) into the ZIF-8 framework. They then utilized microfluidic chips to construct aldehyde-methacrylate-hyaluronan/collagen hybrid hydrogel microfibers that contained IL4@ZIF-8 (Xin et al., 2022). As shown in Figure 10, these neural microfibers not only induced nerve growth but also protected nerve cells by inhibiting inflammatory responses. The tests conducted indicate that the neural microfibers incorporating IL4@ZIF-8 achieved this protection via enhancing the M2 polarization of recruited macrophages and inhibiting the inflammatory response. Additionally, the hydrogel microfibers effectively promoted the growth and differentiation of neuronal cells. In summary, ZIF-8 primarily functions as a drug carrier in neural repair engineering. Its ability to intelligently release drugs in low pH inflammatory environments makes it highly suitable for nerve repair applications.

### 4.3 Vascular tissue scaffold

**FIGURE 11:**
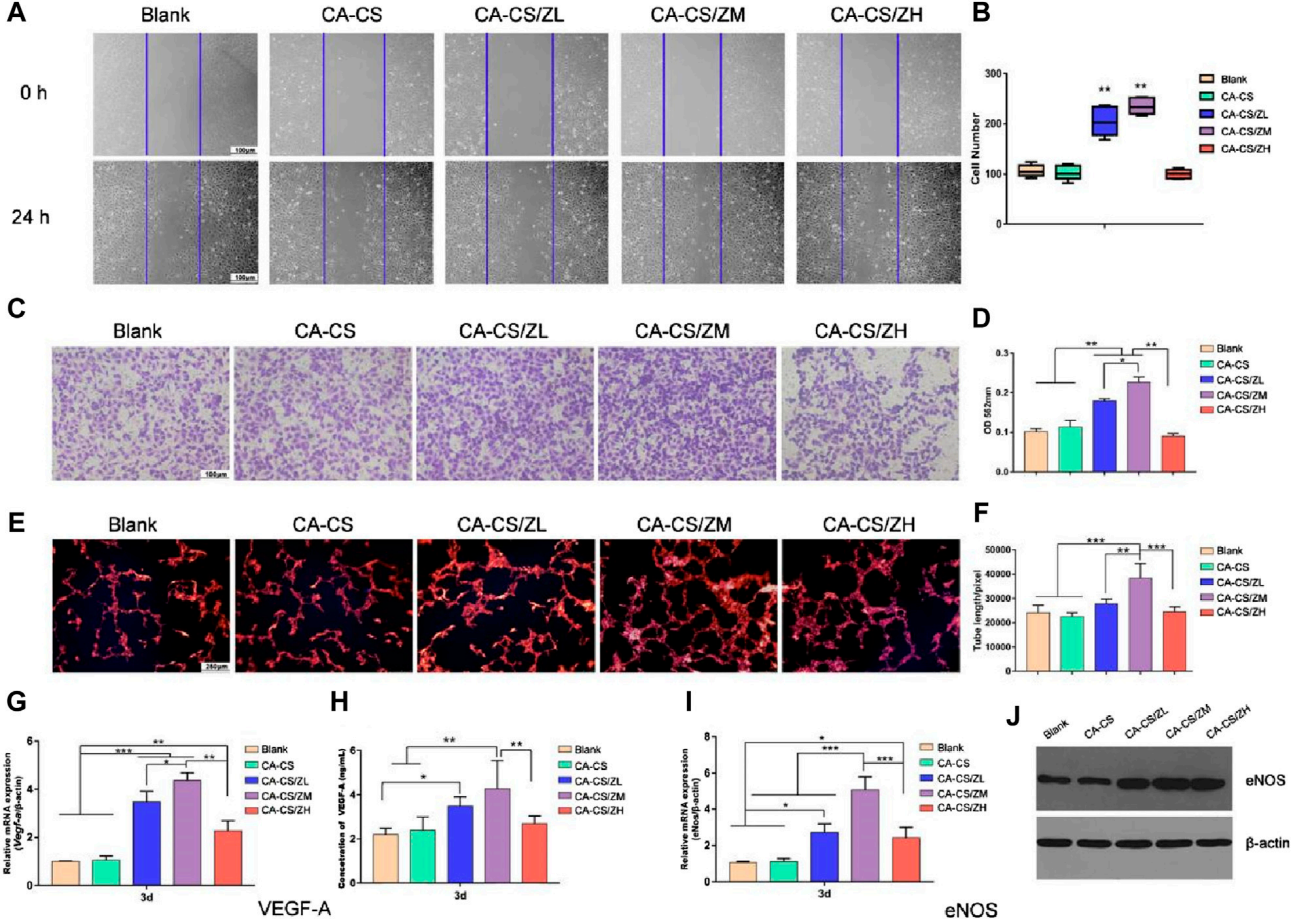
Indirect vascular stimulation test of the scaffold (Liu Y. et al., 2020) **(A)** Microscopic photos and **(B)** scratch test results (Scale bar for 100 μm) **(C)** Crystal violet test results and **(D)** corresponding absorbance reading (at 562 nm) of different experimental groups **(E)** Nuclear staining images and **(F)** corresponding absorbance reading of different experimental groups; Expression levels of **(G)** the *Vegf-a* gene in rBMSCs and **(I)** *eNos* gene in HUVECs of CA-CS and CA-CS/Z hydrogel groups **(H)** Quantification of VEGF-A secretion; **(J)** Results of Western blot on eNOS of each group.

In clinical practice, patients often face various vascular diseases such as myocardial infarction, endothelial disease, and peripheral vascular disease. In cases where their own blood vessels are insufficient for repair, extracorporeal implantation of vascular stents becomes necessary (Cheng et al., 2022). Although allogeneic or xenogeneic vascular grafts can be used as substitutes, immune rejection reactions are a common concern, limiting their practicality. Therefore, the development of artificial vascular stents that mimic the flexibility and ductility of biological blood vessels while avoiding immune rejection reactions is of great interest. In recent research, artificially synthesized biological vascular scaffolds have shown the potential to stimulate the growth and repair of surrounding blood vessels. ZIF-8, with its high drug loading capacity, can effectively release growth-promoting drugs in vascular tissue scaffolds, thus promoting vascular repair and regeneration. In one study, Liu et al. designed a multifunctional hydrogel (CA-CS/Z) by modifying Catechol chitosan (CA-CS) with ZIF-8. This hydrogel, as shown in Figure 11, promotes the secretion of Vascular endothelial growth factor (VEGF) in rat bone marrow mesenchymal stem cells (rBMSCs), ensuring adequate blood supply in areas with bone defects (Liu Y. et al., 2020). In another study, Feng et al. explored the use of nanoscale ZIF-8 as a vector for miRNA delivery, achieving efficient cellular uptake and payload release at a specific intracellular site (Feng et al., 2022). The results demonstrated that the ZIF-8 vector exhibited high loading efficiency and improved cell uptake, enhancing the endosomal escape ability of miRNA. RNA sequencing analysis revealed the upregulation of the MAPK signaling pathway and PID-HIF1-TF pathway in HUVECs transfected with miR-21@ZIF-8. This ultimately led to the promotion of angiogenesis, as demonstrated through *in vitro* and *in vivo* evaluations.

### 4.4 Skin tissue scaffolds

Skin damage caused by burns, accidents, and physiological diseases is the most common tissue defect disease (Davinelli et al., 2018; Lan et al., 2020). Owing to the inability to self-supply, the development of artificial skin is the main means of treating defects. Ideal skin tissue scaffolds should have high liquid absorption capacity, appropriate gas permeability, biocompatibility, and antibacterial properties to protect skin defects from bacterial infection and dehydration (Pang et al., 2020; Patil et al., 2020). However, hydrophilic material is susceptible to bacteria, where hydrophobic material is anti-fouling, though ineffective to release bactericidal agents. Moreover, these functions are difficult to achieve relying solely on a single implant. Hence, an effective solution is to composite functional nanoparticles with biopolymers to prepare functional artificial skin. Based on previous discussions, it is known that ZIF-8 is known as an effective antimicrobial material that can be applied directly to wounds, and it has great potential in constructing functional nanoparticles. For example, Yao et al. designed an omniphobic porous hydrogel membrane loaded with ZIF-8 via a microfluidic-emulsion-templating method (Zhang Y. et al., 2022). Results showed that the membrane was repellent to blood and body fluids though intrinsically hydrophilic. Significantly, the membrane could release ZIF-8 with bactericidal and anti-inflammatory to the defect site, thus inhibiting bacterial invasion. Moreover, *in vivo* study demonstrated that it accelerated wound closure by promoting angiogenesis and collagen deposition. Compared to *in vivo* bone and nerve repair, phototherapy is more suitable for treating skin injuries, particularly those with open wound structures.

## 5 Major challenges and future prospects

The combination of tissue scaffolds and ZIF-8 is a relatively new research area that presents several challenges. Previous discussions have highlighted the importance of assessing the cytotoxicity of composite scaffolds before their application. While the dose range of nanoparticles has been studied, further investigation is needed to understand the release behavior of ZIF-8 in tissue scaffolds, especially in the context of biodegradable scaffolds. A critical bottleneck is the stability and release behavior of ZIF-8 within the scaffolds, particularly considering the acidic degradation products of polyester-based biopolymers like PLLA, PGA, and PLGA. The degradation behavior of ZIF-8 will be affected by the pH changes in the local environment, which, in turn, can impact the degradation of the tissue scaffold. Therefore, establishing a dynamic degradation pattern between ZIF-8 and tissue scaffolds is necessary. Additionally, different process parameters can influence the properties of composite scaffolds, such as porosity, molding quality, and biomechanical properties, based on the agglomeration characteristics of nanoparticles and the interface force between ZIF-8 and the biopolymer. It is crucial to develop more sustainable and accurate preparation methods to meet the demands of tissue scaffolds.

In the field of drug and gene transportation and delivery, the combination of tissue scaffolds and MOFs holds promise. The pH-responsive characteristics of ZIF-8 can be advantageous in creating new stimulus-responsive biological hybrid materials. Furthermore, ZIF-8 and its derivatives have potential applications in areas such as biological imaging and biosensors. For example, Kumar et al. developed a curcumin-immobilized metal-organic framework-based fluorescent nanoprobe capable of detecting various monovalent and divalent metal ions, with selective detection of Fe(II) (Kumar et al., 2021). Leme et al. designed a label-free electrochemical biosensor based on ZIF-8 for monitoring protein-protein interactions (Trino et al., 2021). In conclusion, ZIF-8 shows great promise in various fields and requires further development and exploration.

## 6 Concluding remarks

ZIF-8 and derivatives provide new insights for nanomedicine due to its unique features, which include high surface area, pH responsive dissolution and ease of functionalization. In order to explore the feasibility of ZIF-8 in the field of tissue regeneration, this review conducts a comprehensive discussion from the perspectives of material synthesis, performance testing, and the construction of multifunctional nanosystems, and then reports on its latest application progress. Obviously, versatile nanoplatform based on ZIF-8 brings many benefits to tissue regeneration, including the construction of a sustained-release drug delivery system, a programmed drug delivery system, and the establishment of a phototherapy nanoplatform. Some key-enablers are listed below.

• The major concern limiting the use of ZIF-8 and its derivatives in tissue regeneration is its unpredictable toxicity. Hydrothermal synthesis is a relatively safe way from chemical synthesis approaches standpoint, which effectively avoids the use of toxic solvents. Besides, the dosage, particle size, and surface characteristics of the powder also affect cell compatibility. Therefore, clarifying the dosage and size range of nanoparticles is the key to ensuring their application.
• The ability to load drugs and the pH responsive dissolution of ZIF-8 provide a pathway for the sustained release and responsive release of drug. In this case, the action time of the drug has been significantly improved, and some drugs with unstable performance have more room for development. More importantly, the treatment can be specific through the difference between the physiological microenvironment of normal cells and damaged cells. These features are well attractive for practical clinical treatment.
• The functional groups of Hmim (ex., amino groups and carboxylic acid) affords anchoring sites for surface functionalization of ZIF-8 and for introducing a ternary nanomaterial (graphene oxide, Mesoporous silica, ionic liquids), thus expanding its application capabilities.
• Currently, ZIF-8 has been preliminarily applied in tissue engineering scaffolds, including bone scaffolds, skin scaffolds, vascular scaffolds and skin tissue scaffolds. After the incorporation of the nanosystem constructed with ZIF-8, the tissue engineering scaffold not only has stable support performance, but also has multiple functions such as antibacterial, promoting cell regeneration and anti-tumor.
